# Imaging Cholinergic Receptors in the Brain by Positron Emission Tomography

**DOI:** 10.1021/acs.jmedchem.3c00573

**Published:** 2023-08-16

**Authors:** Jing-Jing Zhang, Hualong Fu, Ruofan Lin, Jingyin Zhou, Achi Haider, Weiwei Fang, Nehal H. Elghazawy, Jian Rong, Jiahui Chen, Yinlong Li, Chongzhao Ran, Thomas L. Collier, Zhen Chen, Steven H. Liang

**Affiliations:** † Jiangsu Co-Innovation Center of Efficient Processing and Utilization of Forest Resources, Jiangsu Provincial Key Lab for the Chemistry and Utilization of Agro-Forest Biomass, Jiangsu Key Lab of Biomass-Based Green Fuels and Chemicals, International Innovation Center for Forest Chemicals and Materials, College of Chemical Engineering, Nanjing Forestry University, Nanjing, Jiangsu 210037, China; ‡ Division of Nuclear Medicine and Molecular Imaging, Massachusetts General Hospital & Department of Radiology, Harvard Medical School, Boston, Massachusetts 02114, United States; § Key Laboratory of Radiopharmaceuticals, Ministry of Education, College of Chemistry, Beijing Normal University, Beijing 100875, China; ⊥ Department of Radiology and Imaging Sciences, Emory University, 1364 Clifton Road, Atlanta, Georgia 30322, United States; # Department of Pharmaceutical, Chemistry, Faculty of Pharmacy & Biotechnology, German University in Cairo, 11835 Cairo, Egypt; ∇ Athinoula A. Martinos Center for Biomedical Imaging, Massachusetts General Hospital, Harvard Medical School, Charlestown, Massachusetts 02114, United States

## Abstract

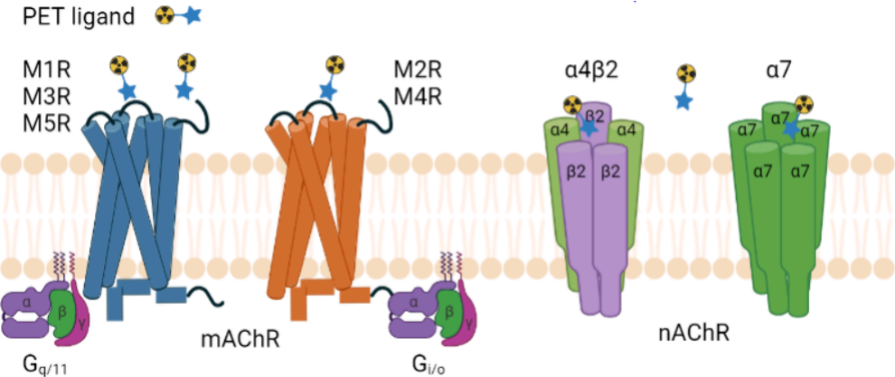

Cholinergic receptors represent a promising class of diagnostic and therapeutic targets due to their significant involvement in cognitive decline associated with neurological disorders and neurodegenerative diseases as well as cardiovascular impairment. Positron emission tomography (PET) is a noninvasive molecular imaging tool that has helped to shed light on the roles these receptors play in disease development and their diverse functions throughout the central nervous system (CNS). In recent years, there has been a notable advancement in the development of PET probes targeting cholinergic receptors. The purpose of this review is to provide a comprehensive overview of the recent progress in the development of these PET probes for cholinergic receptors with a specific focus on ligand structure, radiochemistry, and pharmacology as well as in vivo performance and applications in neuroimaging. The review covers the structural design, pharmacological properties, radiosynthesis approaches, and preclinical and clinical evaluations of current state-of-the-art PET probes for cholinergic receptors.

## Significance

- Cholinergic receptors hold tremendous potential as diagnostic and therapeutic targets given their significant involvement in neurological disorders, neurodegenerative diseases, and cardiovascular impairment.
- Positron emission tomography (PET) has emerged as a noninvasive molecular imaging technique, providing valuable insights into the role of cholinergic receptors in disease development and their diverse functions.
- This review encompasses discussions on structural design, pharmacological properties, radiosynthesis approaches, and both preclinical and clinical evaluations of state-of-the-art PET probes for cholinergic receptors.

## 1 Introduction

**Figure 1:**
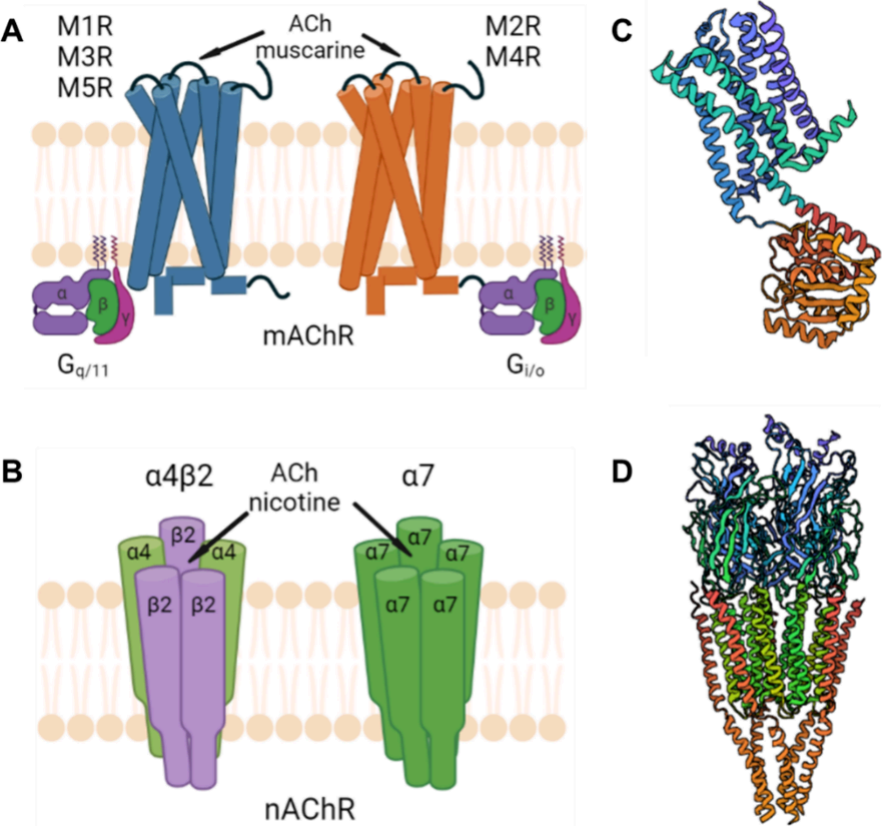
Typical structures of cholinergic receptors. Schematic illustration of (A) mAChRs and (B) nAChRs. Crystal structures of (C) M4 mAChR (PDB 6KP6)^17^ and (D) nAChR (PDB 2BG9).^18^

Acetylcholine (ACh) is an essential neurotransmitter that is involved in various biological processes. Its role in the central nervous system (CNS) includes cognitive processes such as learning, memory, and attention as well as chemical transmission at the neuromuscular junction and autonomic function in the peripheral nervous system (PNS).^1,2^ The action of ACh is mediated through the activation of cholinergic receptors, which can be divided into two classes based on their signaling mechanism: G-protein-coupled muscarinic acetylcholine receptors (mAChRs) and ligand-gated nicotinic acetylcholine receptors (nAChRs; Figure 1).^3^ Among the mAChRs, there are five subtypes (M1, M2, M3, M4, and M5), which are encoded by distinct genes (CHRM1–CHRM5) and have distinct regional distributions in the CNS and peripheral tissues.^4,5^ M1, M4, and M5 mAChRs are predominantly expressed in the CNS and are critical in normal neuronal function. On the other hand, M2 and M3 mAChRs exhibit much wider expression in the periphery and are involved in cardiovascular and secretory processes and gut motility.

On the other hand, nAChRs are composed of pentameric proteins formed by a combination of homomeric or heteromeric subunits among 12 different subunits (α2–10 and β2–4).^6^ In the mammalian brain, the most predominant nAChRs are the homomeric α7 nAChRs and the heteromeric α4β2 nAChRs, both of which are involved in a variety of neuronal processes.^1^ The α7 nAChRs are highly expressed in the hippocampus and cerebral cortex and are associated with cognitive functions such as working memory, long-term memory, and sensory gating.^7^ Studies have shown that expression levels of α7 nAChRs are significantly diminished in schizophrenia patients, and treatment with selective α7 nAChR agonists has been found to alleviate P50 sensory gating impairments and cognitive deficits.^8−11^ Additionally, in Alzheimer’s disease (AD) patients, there is a remarkably decreased level of α7 subunit, suggesting that α7 nAChR agonists may be beneficial in treating cognitive impairments associated with the disease.^12−14^ On the other hand, α4β2 nAChRs have been linked to addiction and hedonic processes and are highly expressed in the hippocampus, cerebral cortex, ventral tegmental area, and substantia nigra.^15^ Varenicline, a partial agonist of α4β2 nAChRs, has been approved for the treatment of smoking cessation.^16^

Positron emission tomography (PET) is a cutting-edge noninvasive imaging modality that has gained widespread use in recent years, particularly in the assessment of biochemical processes and disease staging in real time.^19−21^ Compared to other imaging technologies, PET offers several advantages, such as superior tissue penetration, increased quantification capabilities, and feasibility for clinical translation. The development of a diverse range of PET tracers tailored to visualize specific subunits of cholinergic receptors under normal and pathological conditions has significantly contributed to the advancement of our understanding of the roles and mechanisms of cholinergic receptors in various biological processes, particularly in CNS disorders.^22−26^

In this review, we aim to provide an extensive and current summary of recently discovered PET tracers that selectively target subunits of cholinergic receptors. Additionally, we will discuss their structural diversity, pharmacological properties, and in vivo imaging applications. We hope that this review will facilitate the development of more potent and selective PET ligands targeting cholinergic receptors for clinical use.

## 2 PET Tracers Targeting Muscarinic Acetylcholine Receptors (mAChRs)

mAChRs can be activated by both the endogenous neurotransmitter acetylcholine (ACh) and the exogenous compound muscarine.^1,3,27^ As members of the ACh receptor family and G-protein-coupled receptors (GPCRs), mAChRs play a vital role in regulating cholinergic neurotransmission and various physiological functions. They signal via the activation of intracellular GTP (guanosine triphosphate)-binding regulatory proteins, also known as G-proteins. Each subtype of mAChRs (M1–5) couples to distinct G-proteins. This allows for the modulation of diverse ion channels and other signaling proteins. The M1, M3, and M5 subtypes preferentially couple to G_q/11_ proteins, leading to the activation of phospholipase C and an increase in intracellular calcium levels, thereby promoting an excitatory response. On the other hand, the M2 and M4 subtypes primarily couple to G_i/o_ proteins, resulting in the inhibition of adenylate cyclase and a reduction in intracellular cyclic adenosine monophosphate (cAMP) levels.^28^ The abnormal activity of mAChRs has been linked to the development of several pathological conditions, including cancer, as well as psychiatric and neurological disorders such as AD, schizophrenia, Parkinson’s disease (PD), and drug abuse. These findings highlight the importance of mAChRs in physiological processes and the potential therapeutic implications of targeting these receptors for the treatment of various CNS diseases.^29−32^

### 2.1 pan mAChR PET Tracers

**Figure 2:**
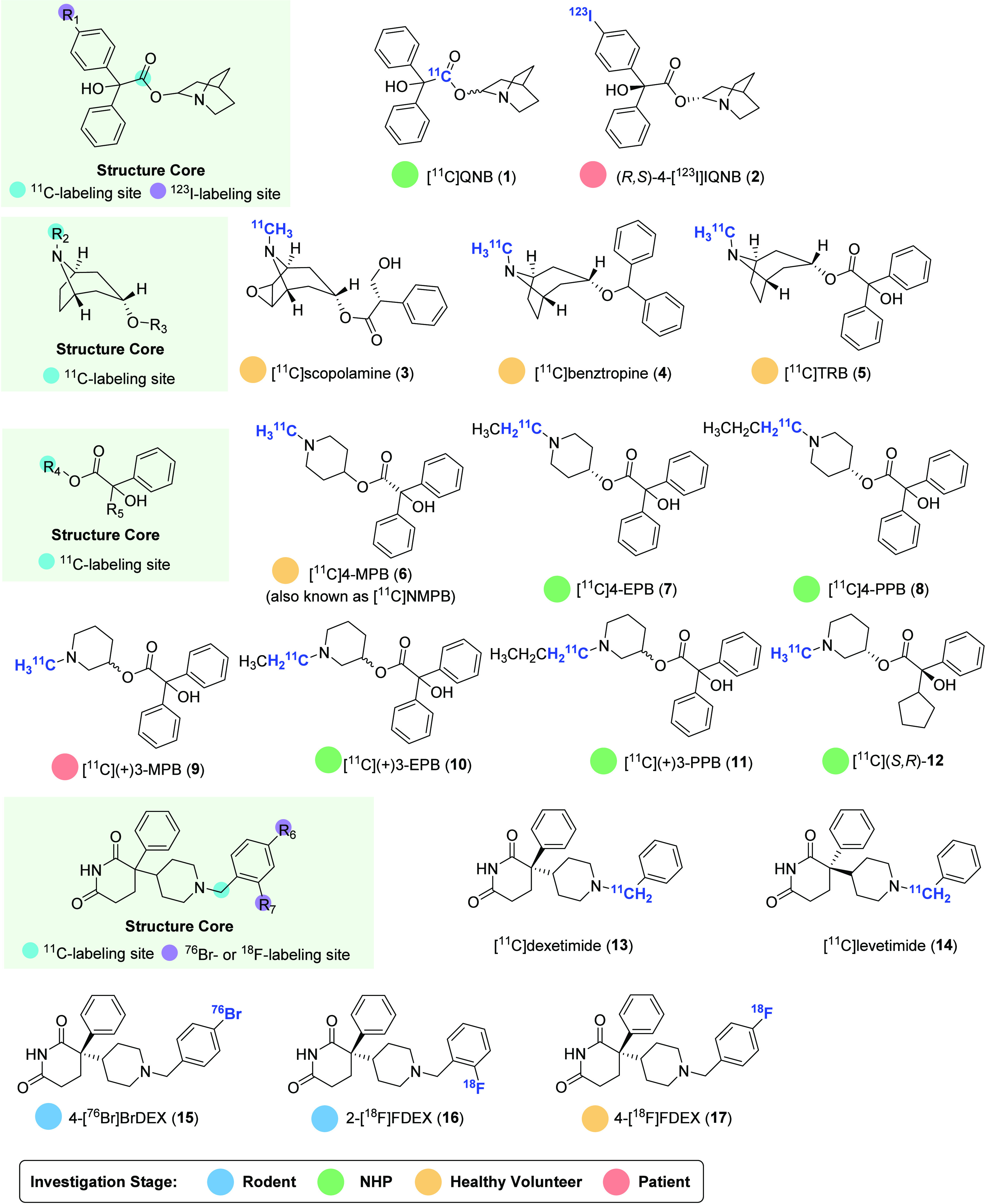
Representative pan mAChR PET ligands.

**Figure 3:**
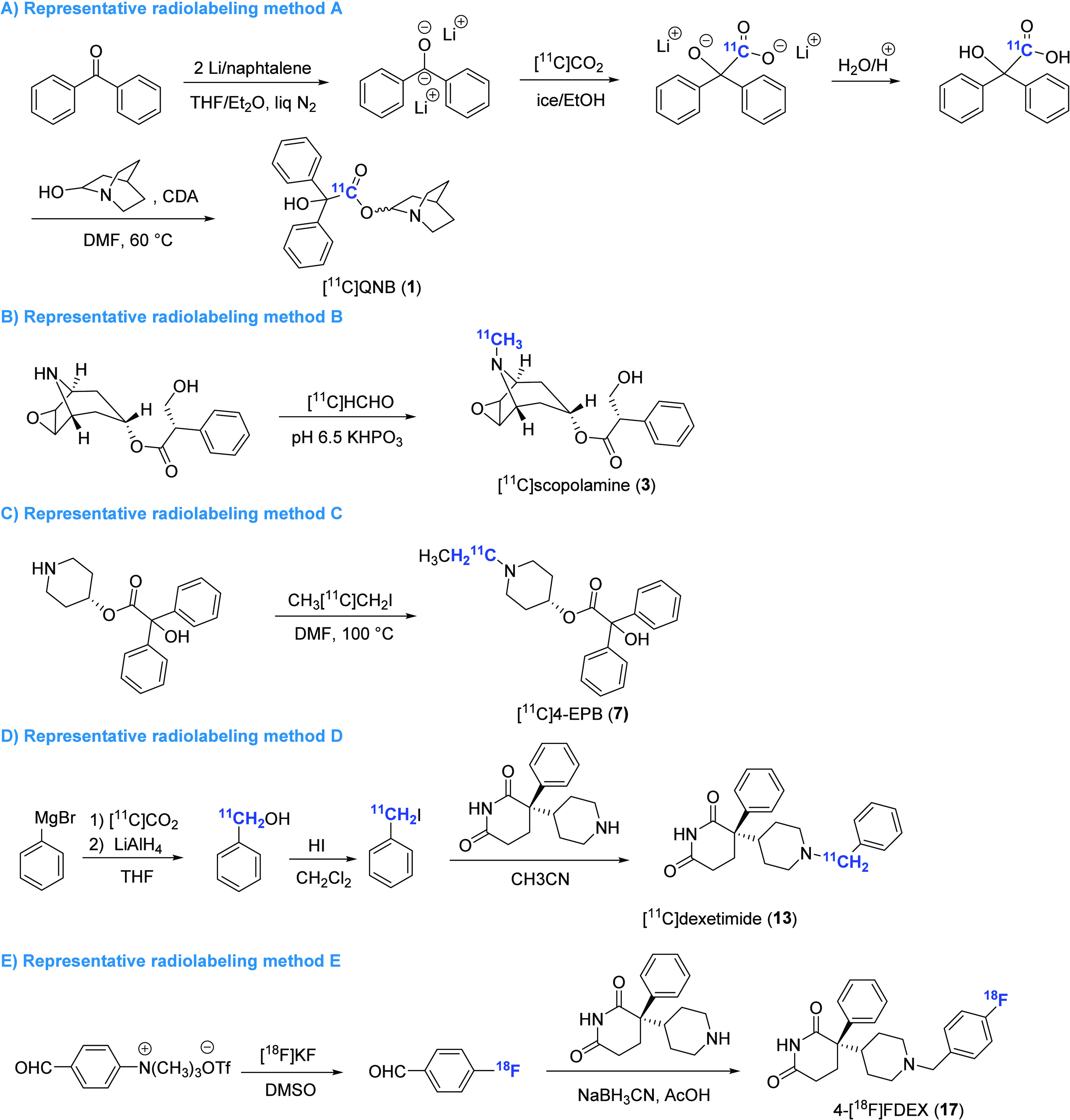
Representative radiolabeling methods for the preparation of pan mAChR PET ligands (CDA, carbonyl diimidazole).

The roles of mAChRs in a variety of brain dysfunctions have motivated numerous studies on the development of mAChR PET ligands. Initially, efforts were primarily focused on the development of pan mAChR agents that lack subtype selectivity. The representative pan mAChR PET ligands and corresponding radiolabeling methods are summarized in Figures 2 and 3, respectively.

Quinuclidinyl benzilate (QNB) is one of the most extensively studied pan mAChR antagonists with a reported dissociation constant (*K*_d_) ranging from 30 to 80 pM for M1–5 mAChRs.^33,34^ Radiolabeling of QNB with carbon-11 led to the discovery of the pan mAChR PET tracer [^11^C]QNB (**1**), which was synthesized by one-pot synthesis of [^11^C]benzylic acid from [^11^C]CO_2_, followed by esterification with 3-quinuclidinol (Figures 2 and 3A).^35^

In vivo PET studies in baboons utilizing [^11^C]QNB (**1**) have shown high accumulation of radioactivity in mAChR-rich regions such as the striatum (1.84%ID/100 mL; %ID = percentage injected dose) and cerebral cortex (2.35%ID/100 mL), while radioactivity rapidly washed out from the cerebellum (approximately 0.5%ID/100 mL), a region known to be deficient in mAChRs.^36^ Pretreatment with dexetimide, a well-established mAChR antagonist, resulted in a significant reduction of radioactivity in the striatum and cerebral cortex (>83%) with no significant changes observed in the cerebellum compared to the control. These findings demonstrate the ability of [^11^C]QNB (**1**) to specifically bind to cerebral mAChRs in primates. However, clinical validation of [^11^C]QNB (**1**) has yet to be reported, and considering the substantial difference in binding affinities between (*R*)-QNB and (*S*)-QNB (a factor of 100), further evaluation of the more active enantiomer [^11^C](*R*)-QNB is warranted.

Despite the focus of this review on PET ligands for mAChRs, an exception was made to discuss radioiodinated QNB due to its significance in the development of mAChR ligands. In particular, radiolabeling of an analogue of (*R*)-QNB with iodine-123 produced two enantiomers.^37−39^ The enantiomer of (*R*)-QNB with higher binding affinity was obtained through chiral resolution and was initially assigned as (*R*,*R*)-4-[^123^I]IQNB.^40^ However, it was later correctly designated as (*R*,*S*)-4-[^123^I]IQNB (**2**).^41^ To date, (*R*,*S*)-4-[^123^I]IQNB (**2**) remains the most extensively investigated mAChR radioligand in clinical studies.^22^ Initial evaluation of (*R*,*S*)-4-[^123^I]IQNB (**2**) with single-photon emission computed tomography (SPECT) imaging indicated significant differences in cerebral radioactivity between AD patients and healthy controls.^42,43^ In healthy individuals, high radioactivity levels were observed in the regions such as the occipital cortex, insular cortex, putamen, and basal ganglia, while the cerebellum revealed an extremely low radioactivity uptake. The regional pattern of (*R*,*S*)-4-[^123^I]IQNB (**2**) correlated well with the known distribution of mAChR in the human brain. However, in AD patients, a significant reduction of radioactivity was observed in the frontal or posterior temporal cortex compared to healthy controls, indicating defects in mAChR in these areas. Autoradiography studies of (*R*,*S*)-4-[^123^I]IQNB (**2**) demonstrated alterations of mAChRs in postmortem brain tissues from individuals with dementia with Lewy bodies (DLB), AD, or PD and the corresponding controls.^44^ In vivo SPECT imaging studies of (*R*,*S*)-4-[^123^I]IQNB (**2**) in schizophrenia patients showed a remarkably decreased mAChR availability (20–33%) in all brain regions except the pons compared with the age- and gender-matched normal controls.^45^ Apart from the applications in investigating mAChR availability in healthy and diseased subjects,^45−47^ (*R*,*S*)-4-[^123^I]IQNB (**2**) has been used to evaluate the effect of drug treatment. Studies have shown that disease treatment with clozapine, an atypical antipsychotic, led to significantly lower radioactive signals in all cortical regions of interest, indicating lower mAChR availability in these regions.^48^ This result suggested that clozapine had high rates of anticholinergic side effects, which was validated by clinical studies. Despite these promising clinical results, it is noted that (*R*,*S*)-4-[^123^I]IQNB (**2**) suffered from the dependence on blood flow and transport across the blood–brain barrier (BBB), which necessitated two clinical scans and pharmacokinetic modeling to differentiate the parameters of blood flow and transport from receptor density.^49,50^ This complexity along with other considerations has led to the discontinuation of investigations with (*R*,*S*)-4-[^123^I]IQNB (**2**) in recent years.

[^11^C]Scopolamine (**3**) is another pan mAChR PET tracer that has demonstrated high binding affinity (IC_50_ = 0.7 nM). It was synthesized in 1988 through phosphite-mediated reductive methylation reactions with [^11^C]formaldehyde (Figure 3B).^51^ Evaluation of [^11^C]scopolamine in human subjects indicated favorable whole brain uptake with an average 3.2%ID between 70 and 90 min p.i.^52^ The radioactive signals were preferentially cleared from brain regions with low mAChR density, such as the cerebellum and thalamus, following initial perfusion-directed radioactivity delivery. In contrast, radioactivity continued to increase during the 2 h scanning period in mAChR-rich areas, such as the cerebral cortex and basal ganglia. However, the cerebral distribution of [^11^C]scopolamine (**3**) at later time points did not align with the known mAChR expression patterns, which has limited its clinical utility.

Benztropine mesylate, also known as Cogentin, is a clinically prescribed anticholinergic drug that is commonly used to alleviate extrapyramidal adverse effects associated with antipsychotic therapy with neuroleptics.^53^ The evaluation of [^11^C]benztropine (**4**) as a PET tracer has demonstrated good brain uptake in mice (3.07%ID/g at 30 min p.i.) and a heterogeneous distribution pattern in baboon and human brains.^54^ The ratios of radioactive levels between the corpus striatum and the cerebellum in baboon and human brains were further determined to be 1.46 and 1.53, respectively. Radioactivity continued to accumulate during the 80 min scanning period in almost all brain regions, except the cerebellum, which exhibited extremely low mAChR density. Pretreatment with scopolamine or benztropine resulted in a remarkable reduction of radioactive levels in all baboon brain regions (35.8% in the corpus striatum, 32.9% in the cortex, and 5.3% in the cerebellum), indicating a favorable binding specificity of [^11^C]benztropine (**4**) toward mAChRs. In addition, metabolic analysis of [^11^C]benztropine (**4**) in human plasma has demonstrated reasonable in vivo stability with 83% parent fraction at 30 min post tracer injection. Despite these promising results, the clinical application of [^11^C]benztropine (**4**) as a mAChR PET tracer has yet to be fully explored.

(+)2α-Tropanyl benzilate (TRB) is a potent mAChR antagonist with high binding affinity (IC_50_ = 0.7 nM),^55^ and its radiolabeling with carbon-11 gave rise to the mAChR radioligand [^11^C]TRB (**5**).^56,57^ Unfortunately, [^11^C]TRB (**5**) was not found to be a promising mAChR radioligand due to its high binding rate and low dissociation rate, making it a less ideal choice for imaging studies.^57,58^

*N*-Methyl-4-piperidylbenzilate (NMPB) is a potent antagonist of mAChR with an IC_50_ value of 1.8 nM and *K*_i_ value of 0.3 nM. The compound is amenable for labeling with carbon-11 to produce [^11^C]NMPB (**6**), also known as [^11^C]4-MPB (**6**).^59^ Similar to [^11^C]benztropine (**4**), radioactivity continued to increase in all human brain regions except the cerebellum during 60 min PET imaging scanning, and the uptake in the frontal cortex reached a peak of ∼17%ID/L at 60 min post tracer injection. Notably, [^11^C]4-MPB (**6**) can be used to measure age-related changes in mAChR activity. By using a compartment model with the radioactivity in the cerebellum as an input function, studies have shown a significant age-related decrease in the specific binding of [^11^C]4-MPB (**6**) in several brain regions, such as the frontal cortex, temporal cortex, and striatum, in both monkeys and humans. The decrease was found to be even more pronounced in humans, reaching approximately 45%.^59,60^ Following the initial studies, two congeners with *N*-ethyl ([^11^C]4-EPB (**7**), *K*_i_ = 0.5 nM) and *N*-propyl ([^11^C]4-PPB (**8**), *K*_i_ = 36 nM)^61^ replacing the *N*-methyl residue in [^11^C]4-MPB (**6**) were synthesized using [^11^C]ethyl iodide and [^11^C]propyl iodide, respectively (Figure 3C).^62^ PET imaging studies of [^11^C]4-EPB (**7**) in nonhuman primates (NHPs) revealed brain kinetics similar to [^11^C]4-MPB (**6**), in which the uptake in all brain regions except the cerebellum gradually increased over a 90 min scanning period. However, PET imaging studies of [^11^C]4-PPB (**8**) revealed different kinetics with the uptake in all brain regions reaching a peak at around 10 min post tracer injection and then gradually decreased over time.

**Figure 4:**
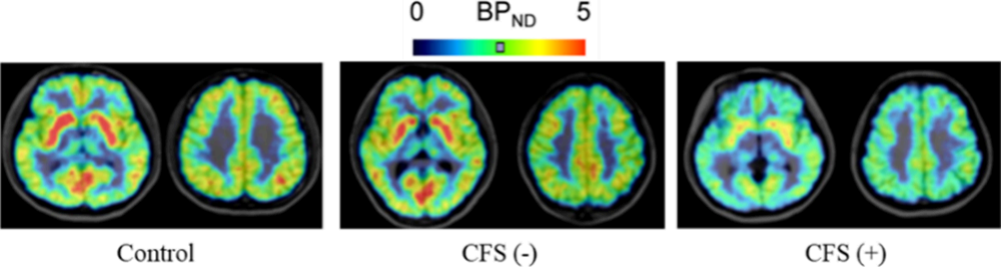
Representative PET images with [^11^C](+)3-MPB (**9**) among normal control and CFS(−) and CFS(+) patients. Reprinted with permission from ref (67). Copyright 2012 PLOS.

Regioisomers of [^11^C]4-MPB (**6**) with regard to the piperidyl position were also investigated, resulting in a pair of enantiomers: the more active [^11^C](+)3-MPB (**9**) (*K*_i_ = 1.7 nM) and its inactive isomer [^11^C](−)3-MPB.^63^ Similar to [^11^C]4-MPB (**6**), kinetic analysis of [^11^C](+)3-MPB (**9**) in conscious monkeys showed an age-related decrease of specific binding in the frontal cortex, temporal cortex, and striatum, reflecting reduced mAChR densities.^64^ By utilizing PET monkey studies with [^11^C](+)3-MPB (**9**), Tsukada and co-workers demonstrated a remarkable positive correlation between mAChR occupancy and oxybutynin or scopolamine-induced cognitive impairment in the striatum, cortices, brainstem, and thalamus.^65,66^ Moreover, the thresholds of mAChR occupancy that could induce cognitive impairment have been proposed to be around 20–30% in the brainstem and around 30–40% in the cortices. It is suggested that a favorable anticholinergic agent should not exceed these thresholds. Furthermore, the ability of [^11^C](+)3-MPB (**9**) to detect mAChR binding alterations in patients with chronic fatigue syndrome (CFS) has also been demonstrated. Studies have revealed that brain binding of [^11^C](+)3-MPB (**9**) in CFS patients with positive tests of CFS(+) antibodies was significantly lower than that in CFS patients with negative results of CFS(−) antibodies and normal controls (Figure 4).^67^

Structural modification of [^11^C](+)3-MPB (**9**) by replacing the *N*-methyl residue with *N*-ethyl or *N*-propyl yielded two analogues [^11^C](+)3-EPB (**10**) and [^11^C](+)3-PPB (**11**).^68^ Radioligands with a longer alkyl chain demonstrated lower binding potential (BP, the ratio of the estimated association rate to the estimated dissociation rate) values with a decreasing order of [^11^C](+)3-MPB (**9**) > [^11^C](+)3-EPB (**10**) > [^11^C](+)3-PPB (**11**). This is likely attributed to a higher dissociation rate constant. Additionally, it was observed that the sensitivity of these radioligands toward ACh had a negative correlation with their binding affinities, and the least potent congener, [^11^C](+)3-PPB (**11**), proved to be the most sensitive to the concentration of endogenous ACh. However, the complex synthesis of [^11^C](+)3-PPB (**11**), which involved the technically challenging preparation of [^11^C]propyl iodide, has hampered its widespread application. Recently, radioligand [^11^C](*S*,*R*)-**12**, an analogue of [^11^C](+)3-PPB (**11**), was developed, which exhibited comparable binding affinity to mAChRs with a *K*_i_ value of 3.5 nM in comparison to [^11^C](+)3-PPB (**11**) with a *K*_i_ value of 7.9 nM.^69^ PET studies of [^11^C](*S*,*R*)-**12** in NHPs have yielded promising results, with high and heterogeneous brain uptake (maximum standardized uptake value (SUV_max_) = ∼8.7 in the caudate and putamen), good specific binding, and a comparable kinetic profile to that of [^11^C](+)3-PPB (**11**). These findings suggest that [^11^C](*S*,*R*)-**12** may have potential as a PET probe for monitoring changes of endogenous ACh concentrations. Further validation through preclinical animal models and clinical studies is required to fully assess the utility of [^11^C](*S*,*R*)-**12** as a PET probe.

Dexetimide (DEX) represents a potent mAChR antagonist with high affinity (IC_50_ = 2.8 nM) and good specific binding (39–73% displaceable binding). Radiolabeling of DEX and its inactive enantiomer levetimide with carbon-11 was achieved via a four-step sequence including Grignard reagent carboxylation, hydride reduction, iodination, and alkylation, thus giving rise to [^11^C]dexetimide (**13**) and [^11^C]levetimide (**14**) in 8% radiochemical yield and reasonable molar activity (0.9 Ci/μmol) (Figure 3D).^70^ However, no further validation has been performed for these two radioligands possibly attributed to the complexity of their synthesis process. In 1996, a ^76^Br-labeled analogue of dexitimide, 4-[^76^Br]BrDEX (**15**), was prepared via electrophilic bromodesilylation of DEX-derived silane with [^76^Br]NH_4_Br. 4-[^76^Br]BrDEX (**15**) exhibited reasonable binding affinity (*K*_d_ = 1.9 nM) and nonspecific binding (23%) in vitro.^71^ Ex vivo biodistribution and in vitro autoradiographic studies revealed high uptake (>0.4%ID/g at 2 h p.i.) in mAChR-rich regions of the rat brain, such as the striatum, hippocampus, and cortex. In contrast, much lower radioactive signals (0.09%ID/g at 2 h p.i.) and rapid and steady clearance were observed in the mAChR-deficient cerebellum.^72^ These findings suggest that 4-[^76^Br]BrDEX (**15**) could be a useful PET radioligand for mAChR imaging; however, further studies are needed to confirm its potential utility.

Two fluorinated analogues of dexetimide, known as 2-fluorodexetimide (2-FDEX) and 4-fluorodexetimide (4-FDEX), were labeled with fluorine-18 to create the first two ^18^F-labeled mAChR PET tracers, namely, 2-[^18^F]FDEX (**16**) and 4-[^18^F]FDEX (**17**).^73−75^ These two radioligands can be effectively prepared by a two-step radiofluorination and reductive amination sequence (Figure 3E).^75^ Biodistribution studies indicated that both 2-[^18^F]FDEX (**16**) and 4-[^18^F]FDEX (**17**) readily crossed the BBB in mice and exhibited a heterogeneous distribution in the brain. mAChR-rich brain regions, such as the striatum (∼9.3%ID/g at 30 min p.i. for 2-[^18^F]FDEX (**16**), ∼5.5%ID/g at 60 min p.i. for 4-[^18^F]FDEX (**17**)) and cortex (∼7.1%ID/g for 2-[^18^F]FDEX (**16**), ∼4.2%ID/g at 60 min p.i. for 4-[^18^F]FDEX (**17**)), had higher radioactivity accumulation, while the mAChR-deficient cerebellum exhibited lower levels (∼0.9%ID/g at 30 min p.i. for 2-[^18^F]FDEX (**16**), ∼0.6%ID/g at 60 min p.i. for 4-[^18^F]FDEX (**17**)).^73^ Blocking studies with dexetimide or atropine resulted in significant blockade of radioactivity uptake in mAChR-rich brain regions (50–95%), indicating good-to-excellent specific binding of [^18^F]2-FDEX (**16**) and 4-[^18^F]FDEX (**17**) toward mouse mAChRs. Further biodistribution studies of [^18^F]2-FDEX (**16**) and 4-[^18^F]FDEX (**17**) in rats also demonstrated similar cerebral distribution patterns with high striatum to cerebellum ratios (12.1 and 12.3 at 3 h p.i., respectively) and high binding specificity.^74^ Despite these promising preclinical results, [^18^F]2-FDEX (**16**) was disadvantaged by in vivo defluorination with high bone uptake (2.4%ID/g), which impeded its further translation into human studies. In contrast, 4-[^18^F]FDEX (**17**) exhibited minimal in vivo defluorination and was recently advanced into human studies, which represents the first and only ^18^F-labeled pan mAChR PET tracer used in humans.^76,77^ PET imaging studies of 4-[^18^F]FDEX (**17**) in humans demonstrated good initial entry into the brain with ∼4.2%ID at 5 min p.i. A heterogeneous distribution was observed with a decreased order of striatum (standardized uptake value (SUV) = ∼6.0 at 4 h p.i.) > cortex (SUV = ∼4.5 at 4 h p.i.) > hippocampus (SUV = ∼3.8 at 4 h p.i.) ≫ thalamus (SUV = ∼3.0 at 4 h p.i.) ≫ cerebellum (SUV = ∼1.3 at 4 h p.i.), consistent with the expression pattern of mAChRs in the human brain. The steady increase of radioactive levels during the 4 h scanning period in mAChR-rich brain regions (striatum, cortex, and hippocampus), a disfavored unsaturable binding characteristic in kinetic modeling, may be partially attributed to the high binding affinity of 4-[^18^F]FDEX (**17**), as reflected from the low *K*_i_ values in the putamen (0.42), frontal cortex (0.27), hippocampus (0.22), and thalamus (0.10). Given these promising results, further validation of 4-[^18^F]FDEX (**17**) in mAChR-related disease models is of paramount importance for future research.

The properties and molecular imaging results of pan mAChR PET ligands discussed in this section are displayed in Table 1.

**Table 1:**
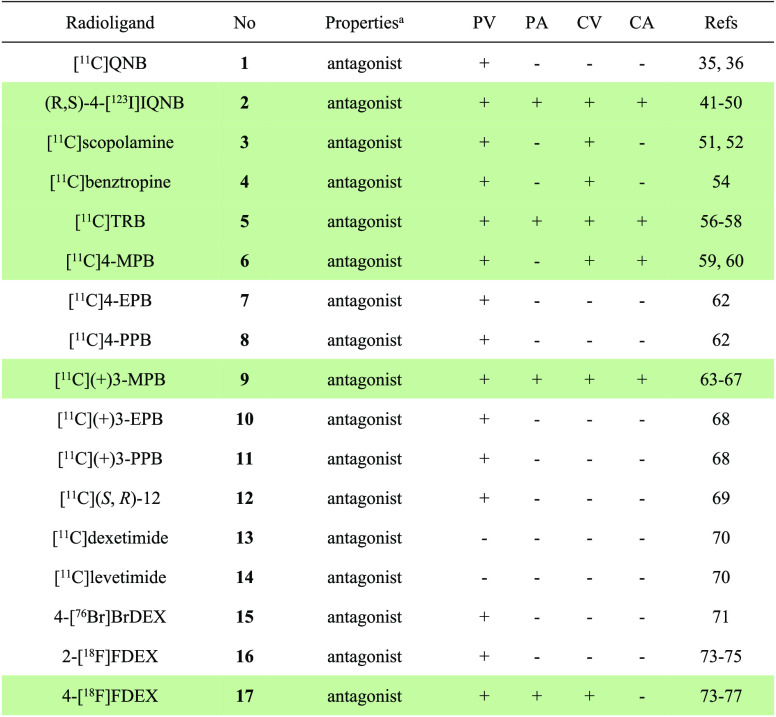
Properties and Molecular Imaging Results of pan mAChR PET Ligands^35,36,41−52,54,56−60,62−71,73−77^

a The definition of antagonist or agonist is based on literature reports. PV, preclinical validation; PA, preclinical application; CV, clinical validation; CA, clinical application; Refs, references. The light green shading indicates the investigation stage of translation into human use.

### 2.2 Subtype-Selective mAChR PET Tracers

**Figure 5:**
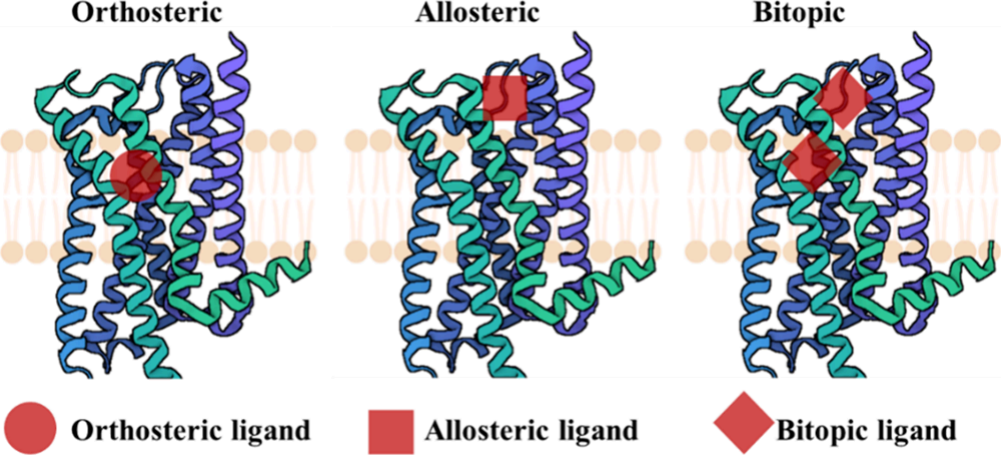
Modes of binding to mAChRs by different classes of ligands.

mAChR ligands can be divided into three categories based on their binding sites: orthosteric, allosteric, and bitopic.^78^ Orthosteric ligands bind to the same site as the endogenous ligand, acetylcholine, while allosteric ligands bind to a distinct site and modulate the activity of the receptor. Bitopic ligands, on the other hand, possess the ability to bind to both the orthosteric and the allosteric sites. As depicted in Figure 5, the orthosteric site of mAChRs has been extensively studied for decades, leading to a thorough understanding of its structure–activity relationships (SARs) and high-affinity binding. However, achieving subtype selectivity at the orthosteric site has proven to be challenging. On the other hand, the allosteric site provides a promising avenue for achieving subtype selectivity. However, modifying certain scaffolds, as done with many orthosteric drugs, may pose challenges in improving the binding affinity.^79^ Notably, bitopic ligands have the potential to target both the orthosteric and the allosteric sites, combining high binding affinity with subtype selectivity. This offers an exciting opportunity for future development of mAChR ligands.

Therefore, compared to pan mAChR ligands, which lack adequate receptor subtype selectivity, the development of allosteric or bitopic mAChR PET tracers with high subtype selectivity has been a major focus in recent years. These subtype-selective tracers enable the specific imaging of certain subtypes of mAChRs, thereby providing valuable information on their expression and distribution in the brain. This, in turn, can aid in the development of more targeted and effective therapeutic approaches for several pathological conditions, such as cancer as well as psychiatric and neurological disorders. To date, subtype-selective PET tracers have been primarily developed for M1, M2, and M4 mAChRs, while rare examples have been reported for M3 and M5 subtypes. This limitation can be attributed to the underdevelopment of nonradiolabeled ligands/drug candidates specifically targeting these two subtypes. Selective PET tracers that target specific mAChRs are herein discussed.

#### Subtype-Selective PET Tracers for M1 Receptor

**Figure 6:**
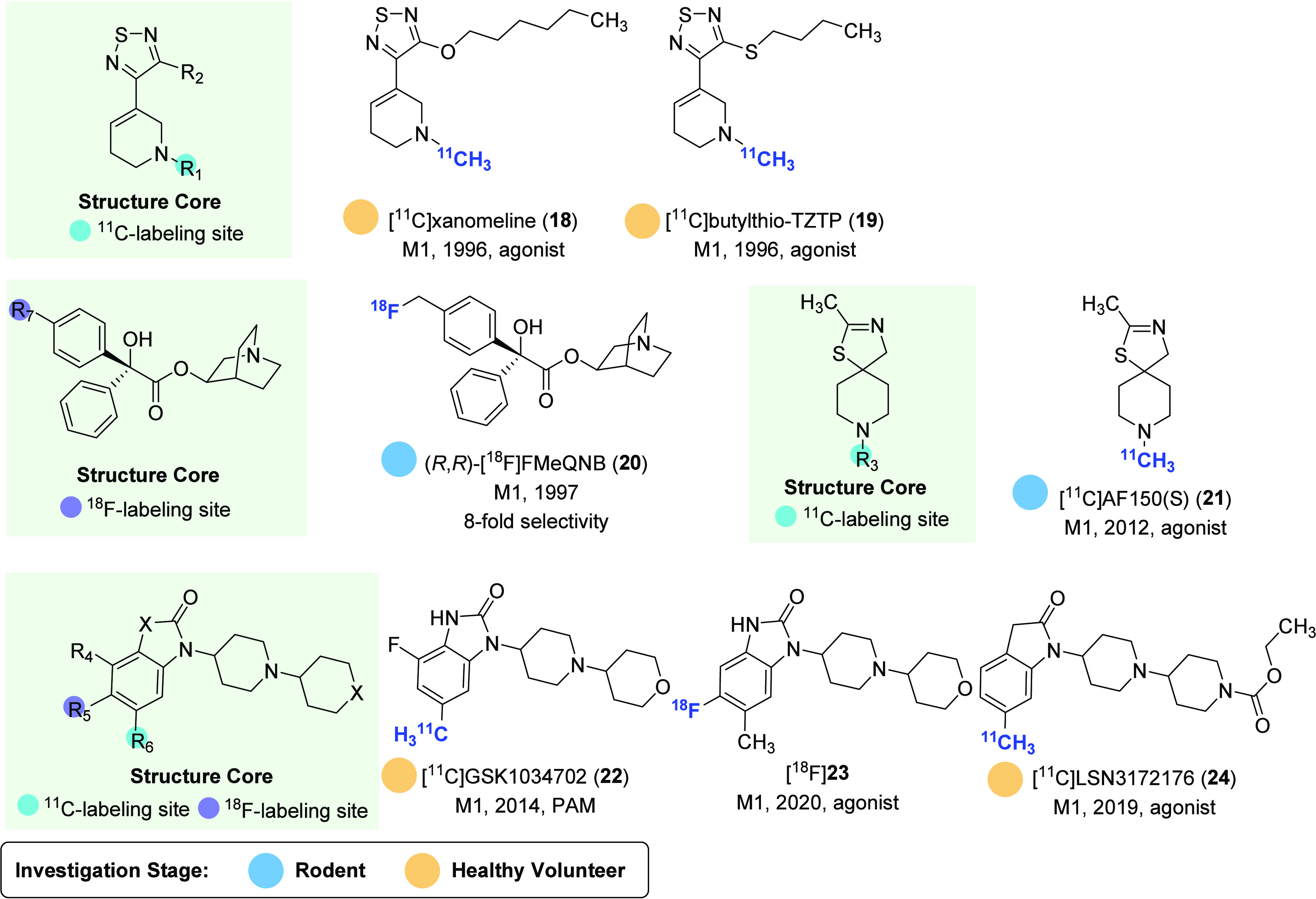
Representative M1-selective PET ligands.

**Figure 7:**
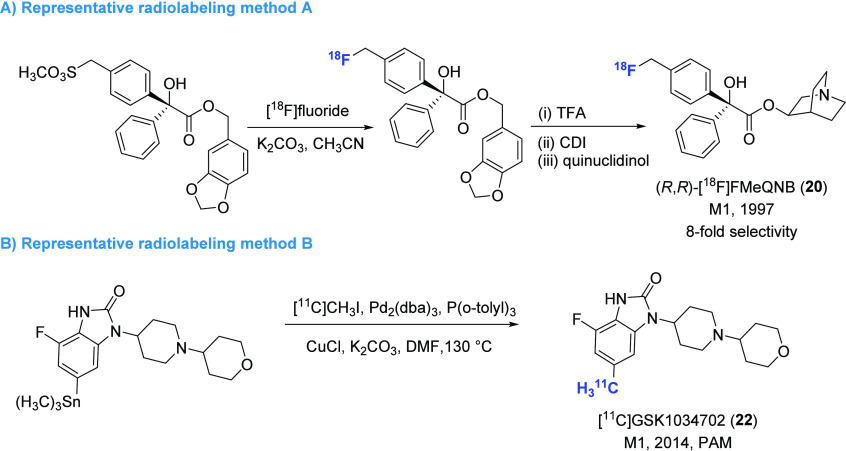
Representative radiolabeling methods for the preparation of M1-selective PET ligands (TFA, trifluoroacetic acid; CDI, carbonyl diimidazole; Pd_2_(dba)_3_, tris(dibenzylideneacetone)dipalladium).

The M1 receptor accounts for up to 60% of the total expression of mAChRs in the CNS and is predominantly found in postsynaptic glutamatergic and striatonigral pyramidal neurons. It is widely expressed in major forebrain areas, including the cerebral cortex, hippocampus, and neostriatum, and plays a crucial role in regulating synaptic plasticity, neurotransmission, and learning and memory. M1 receptor has been implicated in the cognitive decline associated with various CNS pathologies, including AD, and activation of M1 receptor has been shown to improve learning and memory deficits in affected patients.^80^ Several PET ligands that selectively target M1 receptors have been developed, and the representative M1-selective PET ligands and radiolabeling methods are given in Figures 6 and 7, respectively.

The development of M1 receptor PET ligands commenced with the synthesis of the agonist tracers [^11^C]xanomeline (**18**) and [^11^C]butylthio-TZTP (**19**).^81^ These tracers exhibited high affinity (*K*_i_ < 10 nM) to M1 receptors and high selectivity toward M2 receptors.^82^ In preclinical studies, [^11^C]xanomeline (**18**) and [^11^C]butylthio-TZTP (**19**) could efficiently penetrate the BBB of cynomolgus monkeys with ∼10% of injected dose (%ID) and accumulated in the cortex and striatum. However, in healthy volunteers, despite high brain uptake (>5%ID), [^11^C]xanomeline (**18**) had a homogeneous distribution in the brain, while [^11^C]butylthio-TZTP (**19**) only exhibited slightly higher signals in the striatum and neocortex than that of the cerebellum. The lack of desirable PET performance was likely attributed to their poor selectivity toward sigma binding sites.

The stereoselective synthesis of fluorine-containing quinuclidinyl benzilate (QNB) derivatives led to the identification of compounds (*R*,*R*)-FMeQNB and (*R*,*S*)-FMeQNB, which exhibited high affinity (*K*_i_ < 1 nM) and moderate selectivity toward M1 and M2 receptors.^83^ Among these compounds, (*R*,*R*)-FMeQNB demonstrated 8-fold higher affinity for M1 over M2 receptors, with *K*_i_ values of 0.11 and 0.84 nM, respectively. Upon ^18^F labeling (Figure 7A), (*R*,*R*)-[^18^F]FMeQNB (**20**) showed higher signals in the regions of the rat brain known to have high M1 receptor expression, including the cortex, hippocampus, and caudate, albeit at low levels (<0.45%ID/g).^84^ However, the data obtained from coinjection and displacement experiments with (*R*,*R*)-[^18^F]FMeQNB (**20**) were complex and may be influenced by its binding to M1/M2 receptors.

AF150(S) is an agonist of M1 receptors with moderate affinity (*K*_i_ = 390 nM) and high selectivity toward M2 receptors (22 000-fold).^85^ [^11^C]AF150(S) (**21**) was synthesized and subsequently evaluated by in vitro rat brain autoradiography (ARG), ex vivo biodistribution, PET imaging, and radiometabolite analysis.^86,87^ The results of these studies indicated that while [^11^C]AF150(S) (**21**) could rapidly penetrate the BBB, it also had a relatively fast clearance rate from all brain regions. Despite this, apparent specific binding of [^11^C]AF150(S) (**21**) was observed in brain regions that have a high density of M1 receptors, suggesting its potential for imaging the active pool of M1 receptors in vivo. However, the rapid metabolism, hydrophobicity, and moderate binding affinity present significant challenges for its utility in PET studies.

As a positive allosteric modulator (PAM) and agonist, GSK1034702 has moderate affinity (EC_50_ = 7.9 nM) for M1 receptors and high selectivity over other muscarinic receptors (>790 nM). In order to evaluate its utility as a ligand for PET imaging, [^11^C]GSK1034702 (**22**) was prepared through Pd-catalyzed methylation of aryl stannane precursor with a reasonable yield (10% decay corrected, Figure 7B).^88^ The ligand was evaluated in both NHP and human PET imaging.^89^ However, the results of these imaging studies showed that [^11^C]GSK1034702 (**22**) displayed homogeneous and marginal regional heterogeneity in the brain, with regional distribution volume (*V*_T_) values of 3.9 and 4.2 in NHP and the human brain, respectively. These results indicated that [^11^C]GSK1034702 (**22**) was not suitable for imaging M1 receptors in the brain. Nevertheless, the study provided important information to support the clinical development of GSK1034702. More recently, a ^18^F-labeled regioisomer of GSK1034702, [^18^F]**23**, has been developed, but no preclinical evaluation has been reported yet.^90^ The preparation of [^18^F]**23** was achieved via alcohol-enhanced Cu-mediated radiofluorination with a moderate yield of 17%.

LSN3172176 is a bitopic M1-preferring agonist and partial allosteric modulator, which has several similarities with GSK1034702 in chemical structure and has high affinity to M1 receptors (*K*_d_ = 1.5 nM using [^3^H]LSN3172176).^91^ Although the *K*_d_ values for [^3^H]LSN3172176 at M1 (*K*_d_ = 1.5 nM), M2 (*K*_d_ = 0.6 nM), and M4 (*K*_d_ = 0.8 nM) were overlapping in human recombinant cells, the dominant expression of M1 mAChRs in the brain regions may confer its selectivity in vivo. Further experiments using M2 or M4 knockout mice are necessary to validate this postulation in addition to M1 KO target occupancy studies. In PET imaging of rhesus monkeys, [^11^C]LSN3172176 (**24**) exhibited high brain uptake (*V*_T_ 10–18 mL/cm^3^) in M1 receptor-rich regions of the basal ganglia, frontal cortex, and hippocampus but with low *V*_T_ in the cerebellum (poor receptor expression).^92^ The high specific binding of [^11^C]LSN3172176 (**24**) to M1 receptors was strongly demonstrated by the pretreatment experiments with scopolamine (a muscarinic antagonist) and AZD6088 (a M1 receptor-selective partial agonist). When advanced to PET imaging in healthy volunteers, [^11^C]LSN3172176 (**24**) showed selective accumulation in the striatum, neocortical regions, and white matter, and the lowest radioactivity was found in the cerebellum.^93^ Thus, [^11^C]LSN3172176 (**24**) has excellent test–retest reproducibility in estimation of regional acetylcholine concentration variation in living human brain.^94^

Although several M1 radioligands have progressed to clinical trials in healthy volunteers, further efforts are needed to facilitate their translation into the diagnosis and treatment of pathophysiological conditions. It is worth noting that most in vivo evaluated M1 radioligands to date have been limited to ^11^C-labeled compounds. Therefore, future attention should focus on the development and application of more ^18^F-labeled ligands for M1 receptors. This would not only enable more sensitive and accurate imaging of M1 receptors in vivo but also expand the scope of applications of M1 PET imaging in various CNS disorders.

The properties and molecular imaging results of M1-selective PET ligands discussed in this chapter are summarized in Table 2.

**Table 2:**
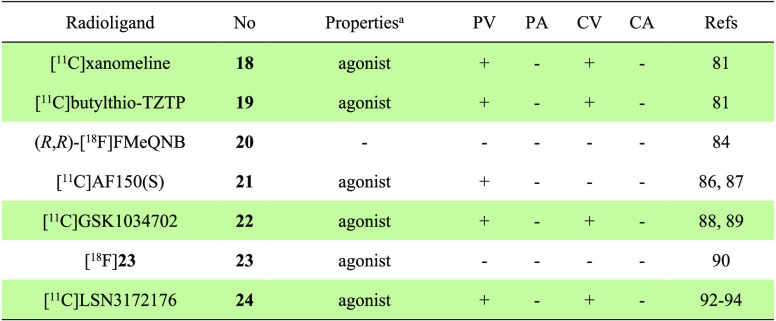
Properties and Molecular Imaging Results of M1-Selective PET Ligands^81,84^,^86−90^,^92−94^

a The definition of antagonist or agonist is based on literature reports. PV, preclinical validation; PA, preclinical application; CV, clinical validation; CA, clinical application. The light green shading indicates the investigation stage of translation into human use.

#### Subtype-Selective PET Tracers for M2 Receptors

**Figure 8:**
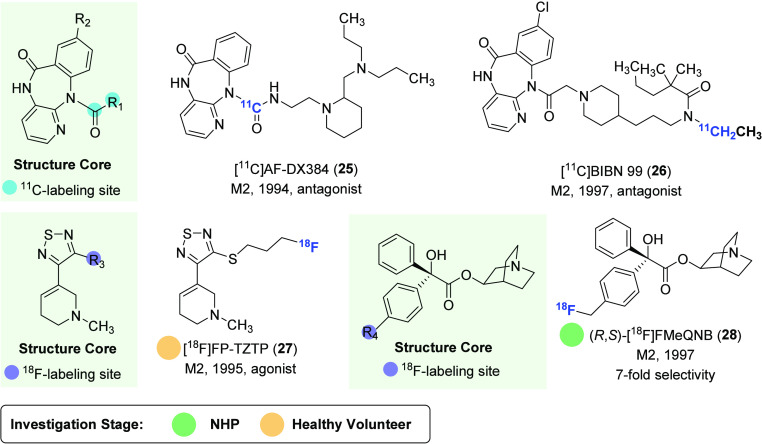
Representative M2-selective PET ligands.

**Figure 9:**
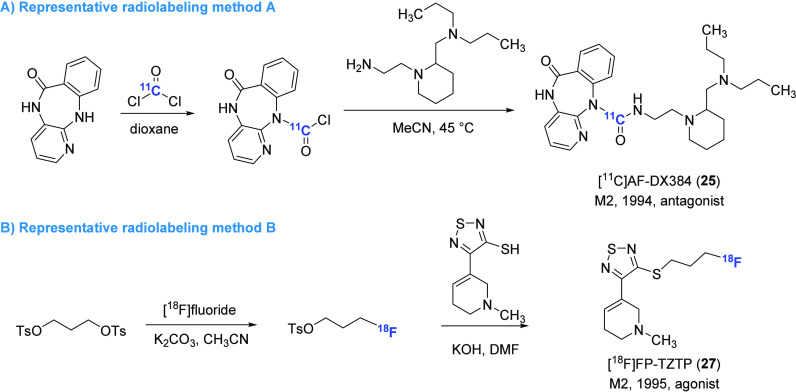
Representative radiolabeling methods for the preparation of M2-selective PET ligands.

The M2 receptor is also widely expressed in the CNS and is among the earliest targets for the development of subtype-selective mAChR radiotracers.^95^ It is abundantly expressed in the basal forebrain, thalamus, neocortex, and hippocampus, especially in noncholinergic terminals in the cortex and hippocampus. M2 receptor also plays a crucial role in learning, recognition memory, and hippocampal plasticity. In rat models, M2 antagonism has been shown to rescue cognitive deficits associated with neurodegeneration.^96^ In light of its significance in the brain, PET ligands that selectively target M2 have been developed and evaluated (Figures 8 and 9).

AF-DX384, an M2 receptor antagonist with high affinities of 0.28 and 28 nM (*K*_i_ values for high- and low-affinity binding sites, respectively), represented a promising starting point for PET tracer development despite its marginal selectivity (*K*_i_(M1)/*K*_i_(M2) = 5).^97^ As a result, [^11^C]AF-DX384 (**25**) was synthesized via urea formation of aniline precursor with [^11^C]phosgene in a high decay-corrected yield of 25–40% (Figure 9A).^98^ However, the low BBB penetration and the presence of brain-permeable radiometabolites have impeded its further application in PET imaging. A structurally similar compound BIBN99 was reported to have moderate affinity for M2 receptors (p*K*_i_ = 7.6 nM) and improved selectivity toward M1 receptors (*K*_i_(M1)/*K*_i_(M2) = 33). [^11^C]BIBN99 (**26**) was obtained with a decay-corrected yield of 26% from [^11^C]ethyl iodide, but to date, no studies have been conducted to investigate its potential use as a PET imaging agent.^99^ Despite sharing similar chemical structures with several reported M1-selective PET ligands, such as [^11^C]xanomeline (**18**) and [^11^C]butylthio-TZTP (**19**), [^18^F]FP-TZTP (**27**) proved to exhibit higher binding affinity for M2 receptors (*K*_i_ = 2.2 nM) in comparison to M1 receptors (*K*_i_ = 7.4 nM).^100^ The selectivity was confirmed through ex vivo ARG studies in knockout mice^101^ as well as in vitro studies in muscarinic receptor-expressing cells and rat brain sections, which suggest that the slower dissociation kinetics of [^18^F]FP-TZTP (**27**) from M2 receptors may be responsible for this selectivity.^102^ Biodistribution results in rats have demonstrated that [^18^F]FP-TZTP (**27**) had rapid uptake and clearance from the brain with relatively uniform accumulation across different brain regions, ranging from 1.1%ID/g in the cortex to 0.6%ID/g in the thalamus at 15 min postinjection.^100^ PET imaging in humans showed that the distribution of [^18^F]FP-TZTP (**27**) using *V*_T_ values in the cortical, subcortical, and cerebellar areas is consistent with M2 receptor expression and correlates with age.^103^ However, additional clinical studies are needed to validate the practical application of [^18^F]FP-TZTP (**27**) in human M2 receptor PET imaging.^104,105^

(*R*,*S*)-FMeQNB has been found to exhibit higher affinity (*K*_i_ = 0.13 nM) for M2 receptors compared to (*R*,*R*)-FMeQNB (**20**) (Figure 6) and demonstrated a 7-fold selectivity over M1 receptors (*K*_i_ = 0.89 nM).^83^ In a study using rat brain, (*R*,*S*)-[^18^F]FMeQNB (**28**) displayed a uniform distribution among different brain regions.^84^ In a coinjection experiment with unlabeled (*R*,*S*)-FMeQNB, the most significant inhibition (97%) of radioactivity uptake was observed in the heart, an organ known to have a high concentration of M2 receptors. Radioactivity was also blocked by 36–54% in all brain regions. Furthermore, in a displacement experiment with unlabeled (*R*,*S*)-FMeQNB at 60 min postinjection of the radioligand, a higher reduction of 30–50% radioactivity was observed in the pons, medulla, and cerebellum, the regions known to contain a high proportion of M2 receptors. These results indicated a reasonable subtype selectivity of (*R*,*S*)-[^18^F]FMeQNB (**28**) in the rat brain. In rhesus monkey, prolonged uptake and retention of (*R*,*S*)-[^18^F]FMeQNB (**28**) was observed in the brain. In a coinjection experiment with unlabeled (*R*,*S*)-FMeQNB, significant radioactivity inhibition was observed in the heart, thalamus, and pons but not in the cerebellum, which also has a high concentration of M2 receptor. (*R*,*S*)-[^18^F]FMeQNB (**28**) was found to degrade rapidly in monkey plasma with less than 5% of the parent compound remaining at 30 min postinjection, which may be likely induced by radiodefluorination in vivo as confirmed by significant bone uptake.

Compared to other mAChR subtypes, the development of M2-selective radioligands has lagged far behind. Most M2 tracers were reported before 1997, and no significant advances have been made in the last two decades. This is partly due to the lack of ligands that exhibit high selectivity between M2 and M1. Given this scenario, future research efforts should focus on the development of highly selective M2 ligands based on recent development of an allosteric binding mechanism (cf. Figure 5) on mAChR ligands.

The properties and molecular imaging results of M2-selective PET ligands discussed in this section are given in Table 3.

**Table 3:**
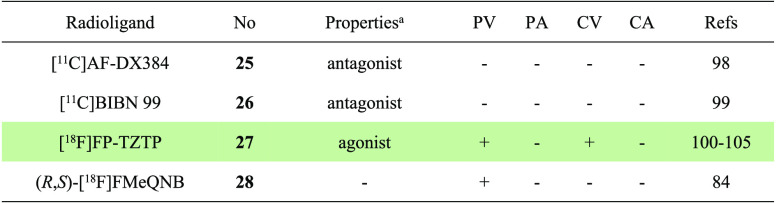
Properties and Molecular Imaging Results of M2-Selective PET Ligands^84,98−105^

a The definition of antagonist or agonist is based on literature reports. PV, preclinical validation; PA, preclinical application; CV, clinical validation; CA, clinical application. The light green shading indicates the investigation stage of translation into human use.

#### Subtype-Selective PET Tracers for M4 Receptors

**Figure 10:**
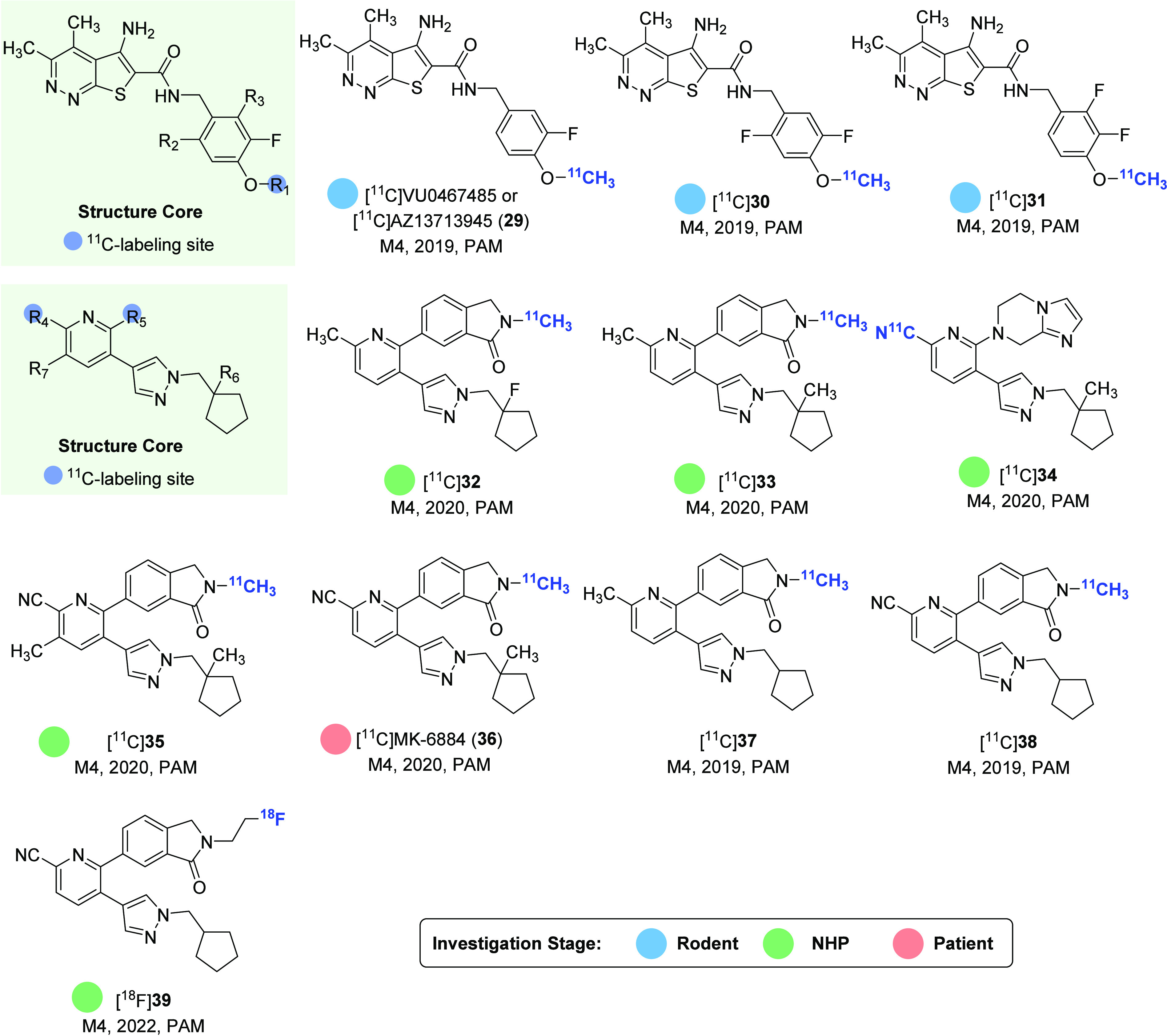
Representative M4-selective PET ligands.

**Figure 11:**
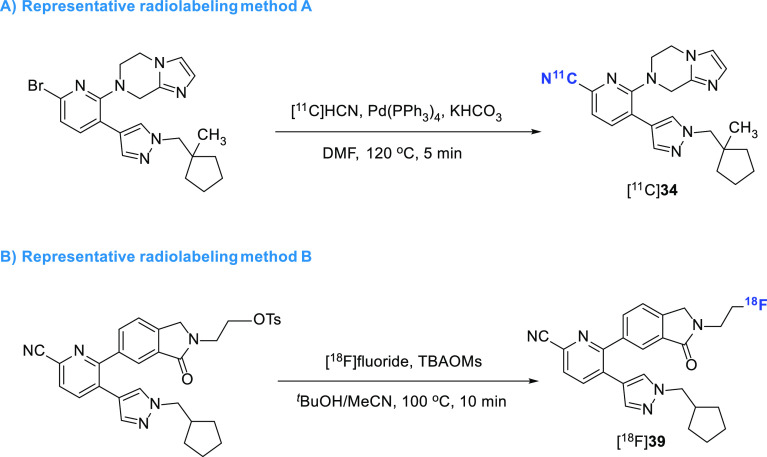
Representative radiolabeling methods of M4-selective PET ligands.

M4 receptor is predominantly expressed in brain regions such as the striatum, hippocampus, and cortex and has been implicated in several CNS disorders, including AD and schizophrenia.^106^ Hence, M4 receptor presents a promising target for the treatment of these diseases. Additionally, M4 is coexpressed with dopamine receptors on striatal projection neurons,^107^ regulating dopamine release and inhibiting dopamine D1 receptor function. This renders M4 as a potential target for the treatment of PD as significant loss of dopamine neuron projecting to the striatum has been implicated in the disease.^108^ Given the critical role of M4 in various brain diseases, the development of subtype-selective M4 PET ligands has been an active area of research, resulting in the design and synthesis of PET tracers with various structural cores for M4 receptors, as shown in Figures 10 and 11.

In 2019, [^11^C]VU0467485 (**29**, also known as [^11^C]AZ13713945) and its two analogues [^11^C]**30** and [^11^C]**31** (Figure 10) were reported as highly selective M4 positive allosteric modulators (PAMs) with EC_50_ values of 78.8, 41.4, and 43.4 nM, respectively.^109,110^ In vitro ARG results in the rat brain sections showed that [^11^C]**31** had the best binding specificity, followed by the medium level of [^11^C]**30**, while [^11^C]VU0467485 (**29**) failed to demonstrate any specific binding. However, [^11^C]**31** was unable to penetrate the BBB in further rat PET imaging. Despite this setback, this work provides a foundation for further chemical optimization in the development of M4-selective PET ligands. Recently, five ^11^C-labeled allosteric M4 radioligands (**32**–**36**, as depicted in Figure 10) were developed from a series of potent PAMs.^111,112^ These radioligands were developed with the intention of being used as tools for the quantification of therapeutic drug occupancy of the M4 receptor via PET imaging. The lead radiotracer [^11^C]**34** was prepared through palladium-catalyzed cyanation reaction of aryl bromide precursor with [^11^C]HCN (Figure 11A). [^11^C]**32** displayed moderate affinity for the human striatum (*K*_d_ = 17.6 nM) but did not show specific binding in the brains of rhesus monkeys. Meanwhile, [^11^C]**33**, [^11^C]**34**, and [^11^C]**35** showed inadequate non-displaceable binding potential (BP_ND_, termed as the ratio of available receptor sites to the equilibrium distribution constant) values of 0.15–0.37 in rhesus monkey brain with PET imaging, which was considered insufficient for accurate quantification of the therapeutic drug occupancy of the M4 receptor via PET imaging.^111^

**Figure 12:**
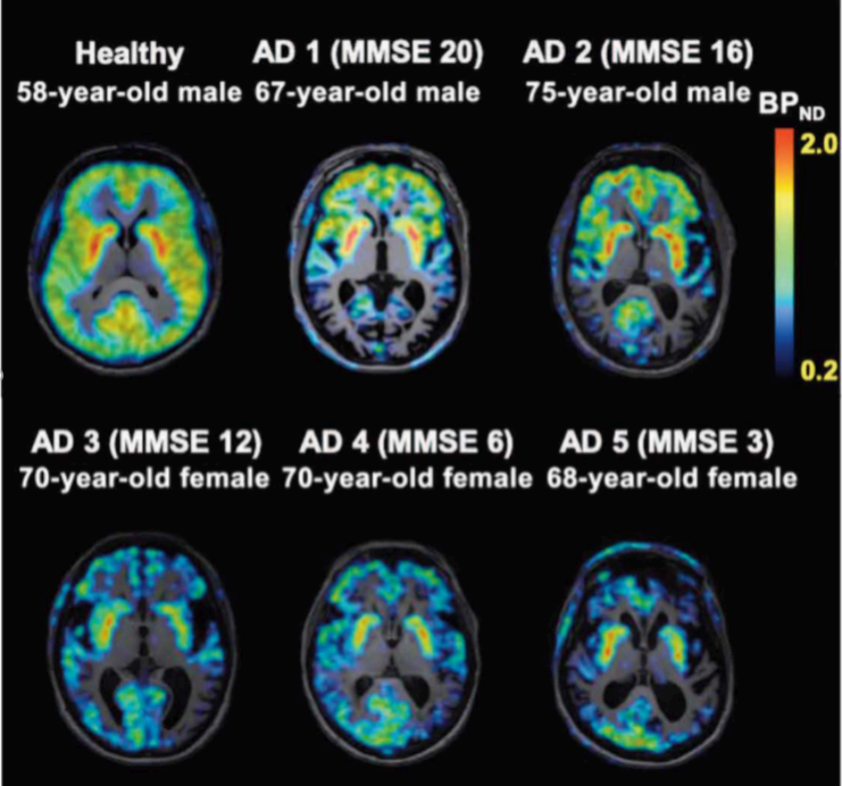
Representative PET images of [^11^C]MK-6884 (**36**) in a healthy adult volunteer taken under baseline conditions and AD patients receiving treatment with donepezil, an acetylcholinesterase inhibitor. Reprinted with permission from (113). Copyright 2022 American Association for the Advancement of Science.

Fortunately, compound MK-6884 was identified as a highly potent and selective PAM of the M4 receptor with high affinity and selectivity (*K*_i_ = 0.19 nM and greater than 3600-fold selectivity over other receptors). In addition, in vitro binding potential values were favorable with *B*_max_/*K*_d_ = 14.4 and 7.8 for monkey and human brain tissues, respectively. In ARG studies using [^3^H]MK-6884, regional binding density was found to be highest in the striatum, followed a decreasing order in the cortex ≈ hippocampus > thalamus ≫ cerebellum in both monkey and human brain sections. Furthermore, the binding signal was significantly enhanced by the orthosteric mAChR agonist carbachol.^113^ In a PET study with rhesus monkeys, [^11^C]MK-6884 (**36**) was found to rapidly penetrate the BBB and accumulate in the striatum with a robust BP_ND_ value of 0.83. In addition, the specific binding of [^11^C]MK-6884 (**36**) to M4 receptors in the brains of rhesus monkeys was confirmed through blocking experiments with another M4 PAM.^111^ Encouraged by these findings, [^11^C]MK-6884 (**36**) was subsequently employed in imaging studies of M4 receptors in healthy volunteers and patients suffering from AD (Figure 12).^113,114^ These studies revealed a significant difference of BP_ND_ values observed between the temporal cortex of healthy elderly participants (0.83 ± 0.15) and AD patients (0.46 ± 0.18). Overall, the development and application of [^11^C]MK-6884 (**36**) as a radioligand highlights its utility in the discovery and evaluation of M4 PAMs as well as the study of M4 receptor changes in neuropathological conditions such as AD.

Most recently, two ^11^C-labeled analogues and one ^18^F-labeled analogue of MK-6884 were also disclosed by our group, thus giving rise to radioligands [^11^C]**37** (EC_50_ = 97.3 nM), [^11^C]**38** (EC_50_ = 37.8 nM), and [^18^F]**39** (EC_50_ = 33.0 nM) (Figure 10).^115,116^ While no biological evaluation was performed for [^11^C]**37** and [^11^C]**38**, preliminary NHP PET study of [^18^F]**39** demonstrated the highest uptake in M4-rich brain regions, such as the putamen and cortical regions, including the prefrontal and temporal cortex. Nonetheless, [^18^F]**39** exhibited a limited overall brain uptake, which impeded its further evaluation.

The development of M4-selective radioligands has faced several challenges, and most of the currently available radioligands have limitations. However, [^11^C]MK-6884 (**36**) has shown promise and has been successfully translated into human use, indicating that further investigation is warranted. Additionally, the use of longer lived isotopes such as ^18^F may help overcome some of the limitations associated with short-lived isotopes and may be a priority for future research in the development of M4 radioligands.

The properties and molecular imaging results of M4-selective PET ligands discussed in this section are shown in Table 4.

**Table 4:**
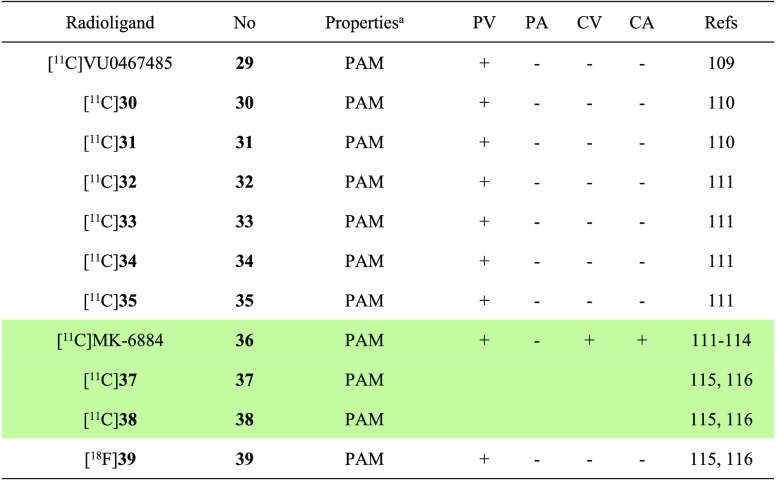
Properties and Molecular Imaging Results of M4-Selective PET Ligands^109−116^

a The definition of antagonist or agonist is based on literature reports. PV, preclinical validation; PA, preclinical application; CV, clinical validation; CA, clinical application. The light green shading indicates the investigation stage of translation into human use.

## 3 PET Tracers Targeting Nicotinic Acetylcholine Receptors (nAChRs)

nAChRs are a type of transmembrane receptor that plays a vital role in fast synaptic transmission in the CNS. These receptors can be activated by both endogenous ligand, ACh, and exogenous ligands, such as nicotine.^6,117,118^ They are also involved in the regulation of processes like cell excitability, transmitter release, and neuronal integration by acetylcholine-mediated innervation, which contributes to their critical role in modulating a wide range of physiological processes, including pain, fatigue, cognition function, sleep, and arousal.^119^ nAChRs are critical in early pre- and perinatal circuit formation and age-related cell degeneration, making them an important target in the pathogenesis of various neurological disorders and neurodegenerative diseases such as AD, PD, myasthenia gravis, and atherosclerosis.^1,120−122^

Neuronal nAChRs are pentamers of heteromeric or homomeric combinations of α (α2-α10) and β (β2-β4) subunits, which gives rise to multiple nAChR subtypes.^119^ In this section, we will focus specifically on the development of PET tracers for the two most expressed nAChR subtypes found in mammalian brains: α7 and α4β2.

### 3.1 PET Tracers for Imaging α7 nAChRs

**Figure 13:**
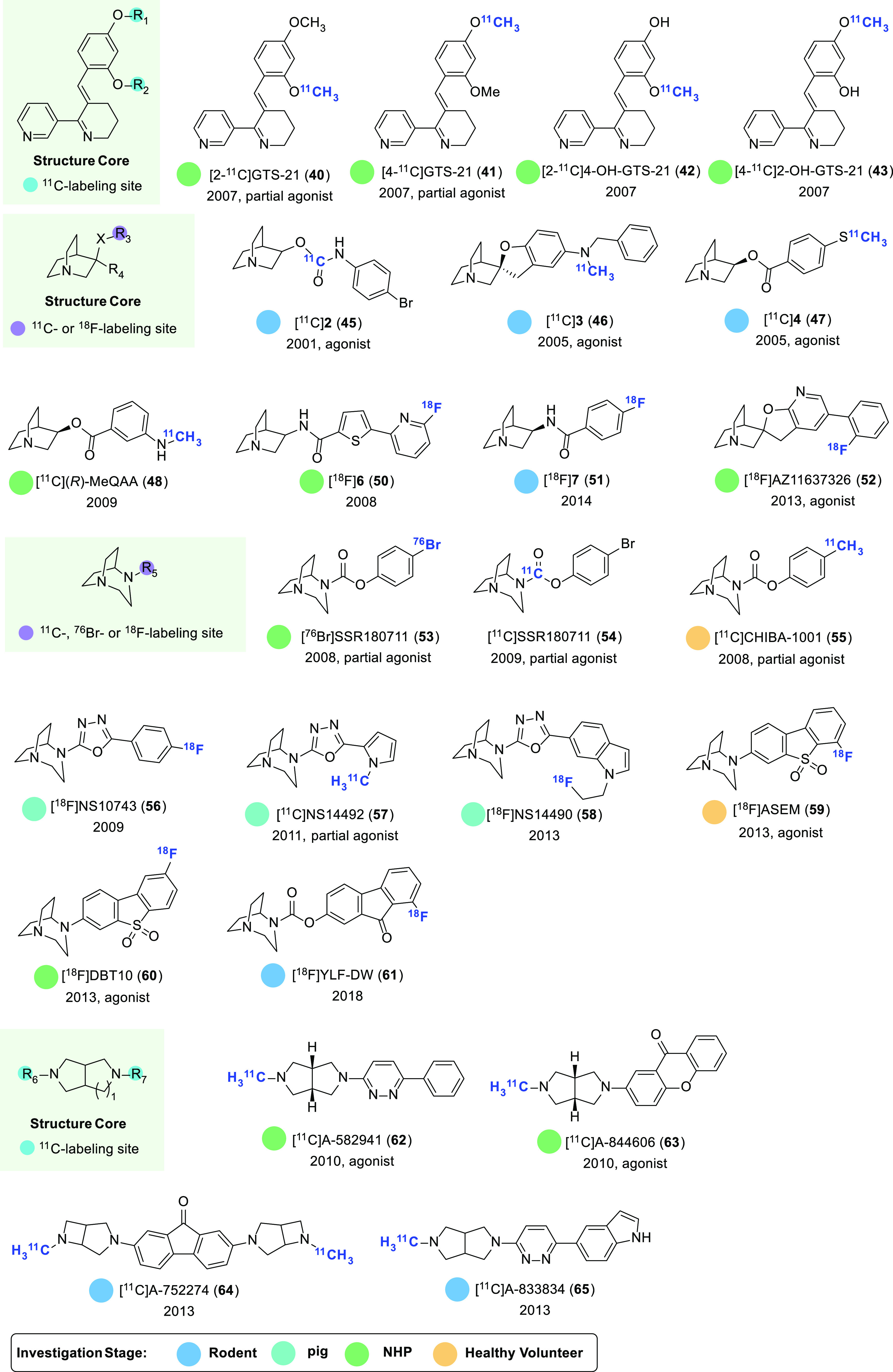
Representative α7 nAChR-selective PET ligands.

**Figure 14:**
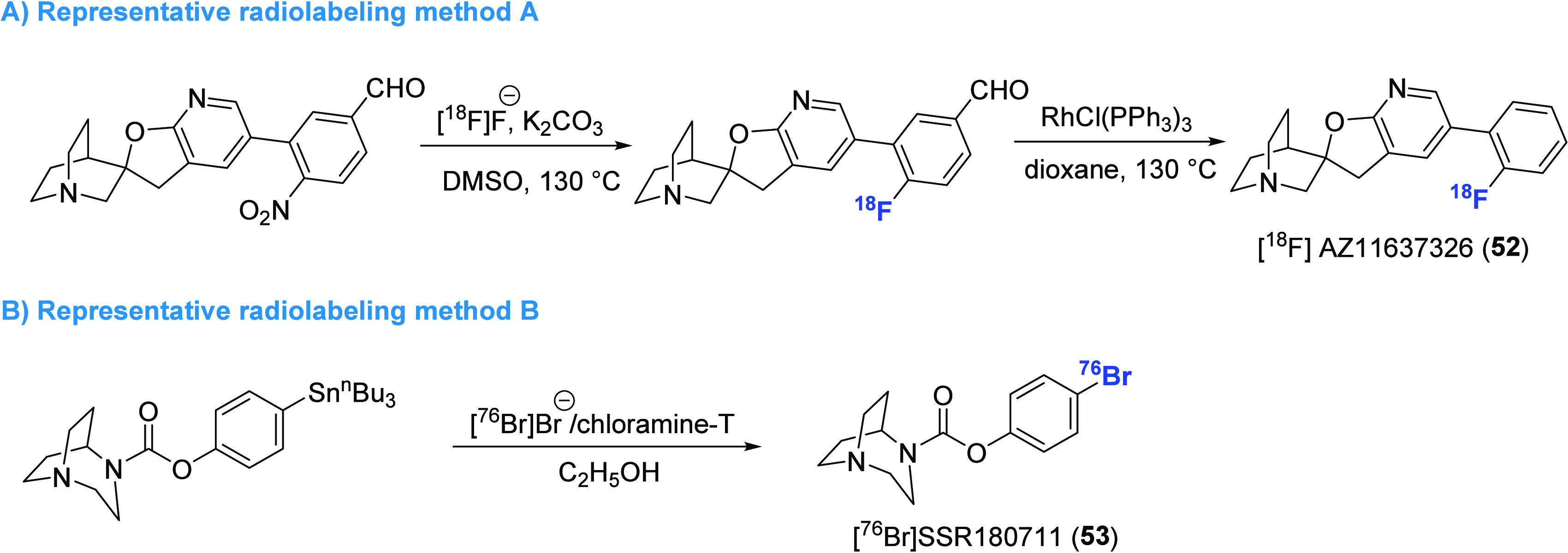
Representative radiolabeling methods for the preparation of α7 nAChR-selective PET ligands.

The α7 nAChR subtype is unique as it is a homomeric complex that contains five equivalent agonist-binding sites in the extracellular domain. These receptors are expressed on both pre- and postsynaptic membranes and act as modulators of circuit activity in the brain.^123^ Studies have shown that α7 nAChRs are involved in the pathogenesis of various neurological disorders.^124^ Given their critical role in cognitive function, there has been a surge in research efforts focused on developing potent and selective α7 nAChR agonists and PAMs for treating a wide range of neuropsychiatric disorders and neurodegenerative diseases, including AD, schizophrenia, depression, anxiety, and addiction.^125−127^ Varieties of PET tracers for imaging α7 nAChRs have been designed and synthesized (Figures 13 and 14).

GTS-21, also known as DMXB-A, is a partial α7 nAChR agonist that has been demonstrated to improve memory in multiple animal models.^128,129^ This compound has also been translated into clinical trials for the enhancement of cognition in patients with schizophrenia.^10,130^ In 2007, Kim and colleagues reported the radiolabeling of GTS-21 with carbon-11 at two different positions as well as the radiolabeling of its two in vivo metabolites, thereby creating four radioligands: [2-^11^C]GTS-21 (**40**), [4-^11^C]GTS-21 (**41**), [2-^11^C]4-OH-GTS-21 (**42**), and [4-^11^C]2-OH-GTS-21 (**43**) (Figure 13).^131^ The pharmacokinetic properties of [2-^11^C]GTS-21 (**40**) and [4-^11^C]GTS-21 (**41**) were evaluated in baboons utilizing PET. These studies revealed that both radioligands had good initial brain uptake, reaching a peak of 0.036 and 0.037%ID/cc at 1.4 and 1.7 min post tracer injection, respectively. Additionally, the tracers exhibited a rapid clearance with a half-life of less than 15 min. Further evaluation of another ligand [4-^11^C]2-OH-GTS-21 showed significant uptake in the baboon brain, while [2-^11^C]4-OH-GTS-21 (**42**) had negligible accumulation. These findings suggest that the metabolite 2-OH-GTS-21 may partially contribute to the therapeutic effects of GTS-21.

As the first full α7 nAChR agonist, AR-R17779 (**44**) features a 1-aza-bicyclo[2.2.2]octane scaffold and shows reasonable binding affinity (*K*_i_ = 92 nM) and selectivity toward α7 nAChRs over α4β2 nAChRs (*K*_i_ = 16 μM).^132^ Some derivatives of AR-R17779 (**44**) were patented as α7 nAChR agonists; however, there is no available pharmacological data.^133^ The radiolabeling of one compound in this series with [^11^C]phosgene resulted in radioligand [^11^C]**2** (**45**).^134^ Ex vivo biodistribution studies of [^11^C]**2** (**45**) in rats demonstrated reasonable brain uptake with 0.8–1.2%ID/g among various brain regions. However, no preferential radioactivity accumulation was observed in regions known to be rich in α7 nAChR, such as the hippocampus, colliculi, and pons. Pretreatment studies utilizing nicotine and nonradioactive compound **2** indicated that there was no specific binding of this ligand toward α7 nAChRs. As a result, [^11^C]**2** (**45**) is not a suitable PET ligand for in vivo visualization of α7 nAChRs. In 2005, Pomper and co-workers described a method for the radiolabeling of two potent α7 agonists containing an 1-aza-bicyclo[2.2.2]octane scaffold with carbon-11. These radioligands exhibited high binding affinity and selectivity toward α7 nAChRs over α4β2 nAChRs (*K*_i_ = 0.54 nM and α4β2/α7 > 22 000 for [^11^C]**3** (**46**); *K*_i_ = 5.8 nM and α4β2/α7 = 14 000 for [^11^C]**4** (**47**)).^135^ Notably, the radiosynthesis of [^11^C]**3** (**46**) was achieved in a reasonable radiochemical yield (6.5%) via a reductive amination reaction of the corresponding desmethylated precursor with [^11^C]formaldehyde, which was in situ generated from [^11^C]CO_2_. However, biodistribution studies of these two radioligands in mice revealed limited brain uptake, homogeneous distribution among various brain regions, and minimal receptor blockade, thus impeding their further in vivo applications. Subsequent studies led to the discovery of a potent α7 nAChR PET ligand [^11^C](*R*)-MeQAA (**48**) (*K*_i_ = 41 nM).^136^ Evaluation of the ligand [^11^C](*R*)-MeQAA (**48**) in mice demonstrated high initial brain uptake, reaching a peak of 7.68%ID/g at 5 min post tracer injection. A heterogeneous radioactivity distribution was observed with higher levels in the hippocampus and lower levels in the cerebellum, which is in agreement with the known α7 nAChR density in the mouse brain. Notably, pretreatment with MLA, an α7 nAChR antagonist, resulted in a significant 32% blockade of radioactive signals in the hippocampus, providing evidence for specific binding of the ligand to α7 nAChR.^137^ Follow-up PET imaging studies of [^11^C](*R*)-MeQAA (**48**) in rhesus monkeys also demonstrated regional brain uptake with high levels in the thalamus (approximately 0.033%ID/mL), moderate levels in the cortex, and low levels in the cerebellum, which is consistent with the α7 nAChR distribution in the monkey brain.

A ^18^F-labeled derivative of a potent α7 nAChR agonist **49** (*K*_i_ = 1.3 nM) was also synthesized to afford the radioligand [^18^F]**6** (**50**).^138^ However, PET study of [^18^F]**6** (**50**) exhibited minimal radioactive signal in the baboon brain. Subsequently, [^18^F]**7** (**51**), an analogue of [^18^F]**6** (**50**), was reported to have moderate binding affinity (*K*_i_ = 18 nM, EC_50_ = 506 nM).^139^ Biodistribution experiments in rats demonstrated a low and homogeneous radioactivity distribution (0.028–0.037%ID/g) as well as poor specific binding (5–25% displaceable radioactivity) in the brain.

In 2014, the synthesis of a high-affinity ^18^F-labeled α7 nAChR PET ligand [^18^F]AZ11637326 (**52**) (*K*_d_ = 0.2 nM) was achieved in 3% nondecay-corrected radiochemical yield (RCY) via a two-step fluorination/decarbonylation sequence (Figure 14A).^140^ Despite limited brain uptake in CD-1 mice (<2.80%ID/g), [^18^F]AZ11637326 (**52**) demonstrated some specific binding in rodents, as indicated by lower radioactivity uptake in α7 nAChR null mice than that of the wild-type controls. However, further PET imaging studies in NHPs indicated minimal specific binding for [^18^F]AZ11637326 (**52**).

In addition to the 1-aza-bicyclo[2.2.2]octane scaffold, 1,4-diaza-bicyclo[3.2.2]nonane is also prevalent in several α7 nAChR agonists. One such example is SSR180711, which has been shown to have a favorable binding affinity for rat (*K*_i_ = 22 nM) and human (*K*_i_ = 14 nM) α7 nAChRs, respectively.^141,142^ Radiolabeling of SSR180711 was achieved through two approaches, namely, radiobromination of the corresponding phenyl–stannane precursor with bromine-76 using chloramine-T as oxidant (Figure 14B) and carbamate formation of 1,4-diazabicyclo[3.2.2]nonane with [^11^C]phosgene. These methods provided two radioligands [^76^Br]SSR180711 (**53**)^143^ and [^11^C]SSR180711 (**54**),^144^ respectively. While no biological data was disclosed for [^11^C]SSR180711 (**54**), [^76^Br]SSR180711 (**53**) was evaluated in conscious monkeys, revealing a heterogeneous distribution pattern and favorable uptake in the brain. The highest radioactivity level was detected in the temporal cortex, reaching a peak of ∼0.0135%ID/mL at around 30 min post tracer injection. In contrast, the cerebellum, a α7 nAChR-deficient region, exhibited the lowest radioactive signals. Pretreatment with nonradioactive SSR180711 rendered remarkably decreased uptake of [^76^Br]SSR180711 (**53**) in various brain regions with the exception of the cerebellum, whereas no obvious alteration of radioactivity levels was observed upon the administration of the selective α4β2 nAChR agonist A85380.

Further studies led to the discovery of a SSR180711 analogue, [^11^C]CHIBA-1001 (**55**), as a potent α7 nAChR PET probe.^143^ [^11^C]CHIBA-1001 (**55**) was readily synthesized with excellent radiochemical yield (9.5% nondecay-corrected RCY) and high molar activity (343.7 GBq/μmol) via the palladium-catalyzed coupling of the corresponding tributyl–stannane precursor with [^11^C]MeI. PET imaging studies of [^11^C]CHIBA-1001 (**55**) in the conscious monkey brain revealed a heterogeneous pattern of radioactivity distribution, which correlated with the density of α7 nAChRs in various brain regions.

Similar to [^76^Br]SSR180711 (**53**), [^11^C]CHIBA-1001 (**55**) demonstrated the highest uptake in the temporal cortex, reaching a peak of ∼0.048%ID/mL at around 20 min post tracer injection. The specific binding of [^11^C]CHIBA-1001 (**55**) was supported by the significant blockade in uptake upon administration of the selective α7 nAChR agonists SR180711 and A844606. Furthermore, subchronic administration of the *N*-methyl-d-aspartate (NMDA) receptor antagonist phencyclidine (PCP), which could induce a schizophrenia NHP model, resulted in a decrease of [^11^C]CHIBA-1001 (**55**) binding in various brain regions with a statistically significant difference observed in the frontal cortex. These promising results prompted the translation of [^11^C]CHIBA-1001 (**55**) into clinical studies for the visualization of α7 nAChR in the human brain.^145^ PET imaging studies of [^11^C]CHIBA-1001 (**55**) in humans revealed a heterogeneous brain distribution with *V*_T_ values ranging from 16.6 to 21.6 in various regions. The highest radioactive signal was observed in the thalamus, reaching a peak of 5.5 SUV at approximately 13 min p.i. Notably, in human subjects, [^11^C]CHIBA-1001 (**55**) exhibited comparable uptake in the cerebellum (SUV_max_ = ∼4.7 at around 13 min p.i.) to that in the cortex, which was in contrast to the low radioactivity accumulation in the cerebellum of monkeys. This difference in uptake was likely attributed to its relatively low binding affinity (*K*_d_ = 120–180 nM).^146^

In 2009, a potent ^18^F-labeled 1,4-diaza-bicyclo[3.2.2]nonane-derived PET ligand [^18^F]NS10743 (**56**) was developed with improved binding affinity to α7 nAChRs (*K*_i_ = 11.6 nM, *K*_d_ = 8.99 nM).^147,148^ Ex vivo biodistribution studies of [^18^F]NS10743 (**56**) in mice indicated good BBB permeability with a peak brain uptake of 4.83%ID/g at 5 min p.i. The target specificity was also validated through preinjection with the selective α7 nAChR agonist SSR180711, which resulted in 28% reduction of radioactive signals in the mouse brain. In vivo PET imaging studies of [^18^F]NS10743 (**56**) in juvenile pigs also demonstrated good brain uptake with high radioactive signals in α7 nAChR-rich regions, such as the hippocampus, thalamus, colliculi, and temporal cortex.^149^ The highest uptake was observed in the colliculi, with a SUV_max_ of 2.48, while the olfactory bulb exhibited the lowest radioactivity level (SUV_max_ = 1.53 at 7 min p.i.). To further evaluate the specificity of [^18^F]NS10743 (**56**), preadministration of the α7 nAChR-selective ligand NS6740 was conducted, which decreased the binding of [^18^F]NS10743 (**56**) by 16–35% based on BP_ND_ (the ratio of specifically bound radiotracer to that of nondisplaceable radiotracer) throughout the brain, except for the olfactory bulb. These results suggest that while the binding of [^18^F]NS10743 (**56**) to α7 nAChR is reasonable, it may not be sufficiently specific in vivo.

Another high-affinity and selective α7 nAChR partial agonist NS14492 was radiolabeled with carbon-11 to provide the radioligand [^11^C]NS14492 (**57**) (*K*_i_ = 2.2 nM for α7 nAChRs; *K*_i_ = 2.8 μM for α4β2 nAChRs).^150^ In vivo evaluation in Danish Landrace pigs indicated that [^11^C]NS14492 (**57**) could readily penetrate the BBB with high radioactive signals in the thalamus and cerebral cortex, moderate signals in the striatum, and relatively low signals in the cerebellum, which was in accordance with the distribution of α7 nAChRs in the pig brain. Notably, the radioactive signals were found to be dose-dependently blocked by preadministration of NS14492 and SSR180711, resulting in up to 81% reduction, supporting that [^11^C]NS14492 (**57**) selectively binds to α7 nAChRs. These results demonstrate that [^11^C]NS14492 (**57**) is a promising radioligand for imaging of α7 nAChRs in the brain.

Recently, [^18^F]NS14490 (**58**) was developed as a ^18^F-labeled α7 nAChR PET ligand with high binding affinity and selectivity (*K*_i_ = 2.5 nM for α7 nAChRs; *K*_i_ > 1 μM for α4β2 nAChRs).^151^ However, subsequent in vivo evaluation revealed limited brain uptake of the radioligand. Ex vivo biodistribution studies in mice demonstrated a low brain uptake level of only 0.16%ID/g at 5 min p.i.^152^ Similarly, in vivo PET imaging studies in pigs revealed a SUV_max_ of 0.54.^153^ These results indicate that [^18^F]NS14490 (**58**) may not be an ideal radioligand for in vivo mapping of cerebral α7 nAChRs.

Recently, two potent α7 nAChR agonists ASEM (also known as JHU82132) and its para isomer DBT10 (also known as para-ASEM or JHU82108) were radiolabeled with fluorine-18 to generate radioligands [^18^F]ASEM (**59**) and [^18^F]DBT10 (**60**), respectively.^154−156^ In vitro binding studies revealed that both radioligands exhibited high binding affinity (*K*_i_ = 0.37 nM for [^18^F]ASEM (**59**); *K*_i_ = 1.32 nM for [^18^F]DBT10 (**60**)) and high selectivity (α4β2/α7 = 1370 for [^18^F]ASEM (**59**); α4β2/α7 = 663 for [^18^F]DBT10 (**60**)) for the α7 nAChR subtype. Biodistribution studies in mice further demonstrated that [^18^F]ASEM (**59**) exhibited higher uptake in the brain compared to [^18^F]DBT10 (**60**), reaching a peak of 7.5%ID/g at 5 min post tracer injection.^154,155,157^ Furthermore, a heterogeneous radioactivity distribution was observed for [^18^F]ASEM (**59**) with high levels in the hippocampus, frontal cortex, and colliculus, intermediate levels in the striatum, and low levels in the cerebellum. This distribution pattern aligns with the known α7 nAChR density in the mouse brain. The uptake of [^18^F]ASEM (**59**) could be dose-dependently blocked by pretreatment with the α7 nAChR agonist DMXB-A or SSR180711, indicating good in vivo specific binding. Notably, in a rodent model of schizophrenia, mutant DISC1 mice, the uptake of [^18^F]ASEM (**59**) in the brain was remarkably lower in comparison to control mice, which is in line with postmortem human results.^157^ Further PET imaging studies of [^18^F]ASEM (**59**) in baboons also demonstrated excellent BBB penetration with a peak SUV of 5 at 5 min p.i.^157^ Regional radioactivity uptake was observed with *V*_T_ values ranging from 14 in the cerebellum to 23 in the thalamus. The in vivo specific binding in α7 nAChR-rich NHP brain regions was determined to be approximately 80–90%, as demonstrated by dose-dependent blockade of radioactivity uptake with α7 nAChR partial agonist SSR180711. Similar results were also obtained in a study evaluating [^18^F]ASEM (**59**) in pigs.^158^

**Figure 15:**
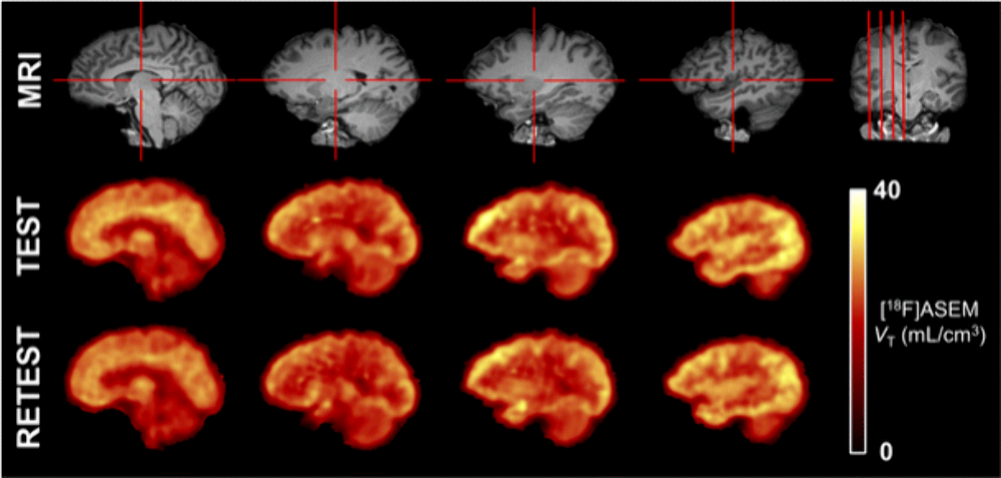
Representative *V*_T_ parametric images from the test and retest scans in a healthy volunteer using [^18^F]ASEM (**59**). Reprinted with permission from ref (160). Copyright 2017 Springer.

Encouraged by the promising preclinical results, the first in human PET studies of [^18^F]ASEM (**59**) were conducted, which revealed high and regional brain uptake (SUV_max_ = 4).^159,160^ Specifically, high levels of radioactivity accumulation were found in the parietal cortex (*V*_T_ = 22), putamen (*V*_T_ = 21.8), thalamus (*V*_T_ = 20.9), temporal lobes (*V*_T_ = 19.7), cingulate (*V*_T_ = 19.6), frontal lobes (*V*_T_ = 19.3), and hippocampus (*V*_T_ = 17.9), and a significantly lower radioactivity level was observed in the corpus callosum (*V*_T_ = 9.9). The regional distribution pattern of [^18^F]ASEM (**59**) in humans matches the postmortem data from humans and NHPs. Furthermore, the potential relationship between α7 nAChR availability and healthy aging was also assessed using [^18^F]ASEM (**59**) (Figure 15).^160,161^ By utilizing Spearman’s rank correlation analysis, the *V*_T_ values of [^18^F]ASEM (**59**) in nine regions of interest in the brain, including the cortical and subcortical regions, were found to be positively correlated with age. Additionally, the tissue volume ratios of six brain regions, including the striatum and five cortical regions (temporal, occipital, cingulate, frontal, and parietal cortices), were negatively correlated with age, which was in accordance with the previous reports on the regional decreases of brain volume over normal aging. Recently, [^18^F]DBT10 (**60**) was also evaluated in NHPs and exhibited relatively lower but still notable brain uptake (SUV = 2.9–3.7) when compared to that of [^18^F]ASEM (**59**).^162^ Given the structural similarity of [^18^F]DBT10 (**60**) and [^18^F]ASEM (**59**), no further validation of [^18^F]DBT10 (**60**) was pursued.

In 2018, Zhang and co-workers developed a new ^18^F-labeled α7 nAChR ligand [^18^F]YLF-DW (**61**) (*K*_i_ = 2.98 nM).^163^ Preliminary biodistribution and PET imaging studies in mice showed favorable brain uptake (8.98%ID/g at 5 min p.i.); however, further validation of the specific binding and evaluation in higher species is necessary. Most recently, [^18^F]YLF-DW (**61**) was applied in identifying vulnerable atherosclerotic plaques in carotid arteries.^164^ Further studies conducted in ApoE^–/–^ mouse models have demonstrated that [^18^F]YLF-DW (**61**) has the potential to be used as a diagnostic tool for the detection of atherosclerotic plaques. In these studies, a significant radioactive signal was observed in the carotid artery of ApoE^–/–^ mice, while no radioactivity was accumulated in the carotid artery of normal control mice. This was consistent with the findings from oil red staining and immunohistochemistry.

In 2010, two α7 nAChR-selective agonists A-582941 (*K*_i_ = 10.8 and 17 nM for rat and human α7 nAChRs; *K*_i_ > 100 μM for α4β2 nAChRs) and A-844606 (IC_50_ = 11 nM for rat α7 nAChRs; IC_50_ > 10 μM for α4β2 nAChRs) were radiolabeled with carbon-11 to provide the radioligands [^11^C]A-582941 (**62**) and [^11^C]A-844606 (**63**), respectively.^165^ Both radioligands effectively crossed the BBB in mice with the brain uptake reaching a peak of 3.92%ID/g for [^11^C]A-582941(**62**) at 5 min p.i. and 7.96%ID/g for [^11^C]A-844606 (**63**) at 30 min p.i., respectively. The good BBB permeability of [^11^C]A-582941 (**62**) and [^11^C]A-844606 (**63**) was also confirmed in studies involving conscious monkeys. However, both radioligands showed a lack of sufficient regional selectivity and specific binding, which hindered their further application. Subsequently, another two ^11^C-labeled radioligands [^11^C]A-752274 (**64**) and [^11^C]A-833834 (**65**) were developed.^166^ Notably, [^11^C]A-752274 (**64**) exhibited very high binding affinity toward α7 nAChR with a *K*_i_ value of 0.092 nM, which is 16-fold higher than that of [^11^C]A-833834 (**65**) (*K*_i_ = 1.53 nM). However, both [^11^C]A-752274 (**64**) and [^11^C]A-833834 (**65**) demonstrated low brain uptake in mice (<1.5%ID/g) and baboons (SUV < 0.3), diminishing their potential utility as PET tracers.

The properties and molecular imaging results of α7 nAChR-selective PET ligands discussed in this section are demonstrated in Table 5.

**Table 5:**
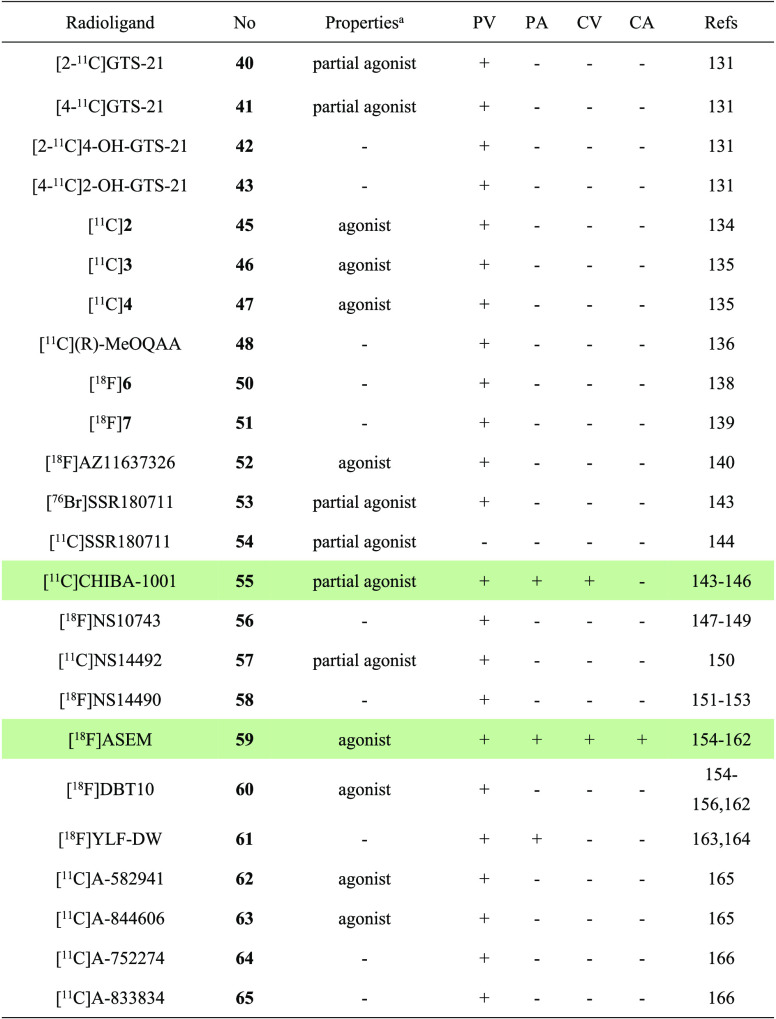
Properties and Molecular Imaging Results of α7 nAChR-Selective PET Ligands^131,134−136,138−140,143−166^

a The definition of antagonist or agonist is based on literature reports. PV, preclinical validation; PA, preclinical application; CV, clinical validation; CA, clinical application. The light green shading indicates the investigation stage of translation into human use.

### 3.3 PET Tracers for Imaging α4β2 nAChRs

**Figure 16:**
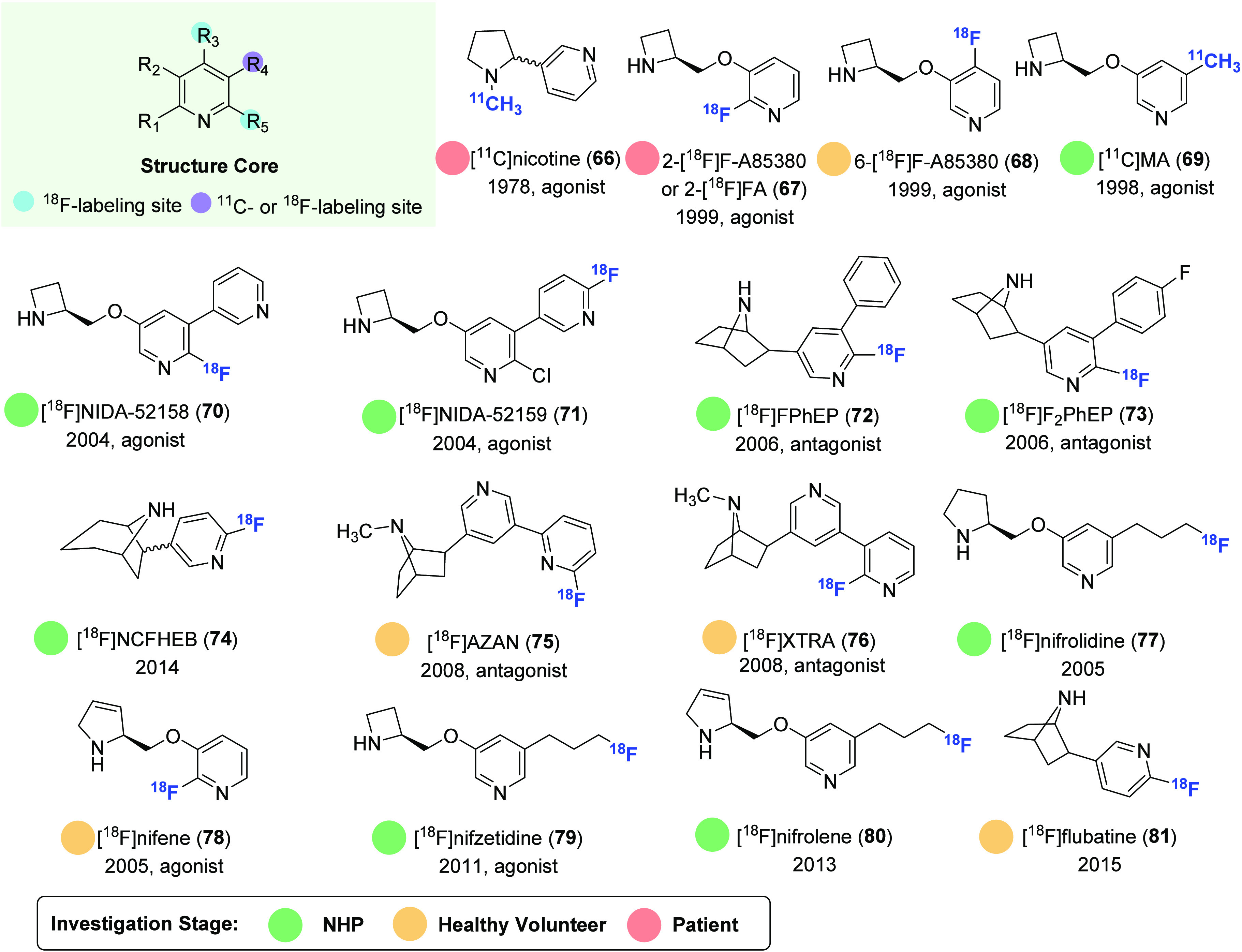
Representative α4β2 nAChR-selective PET ligands.

**Figure 17:**
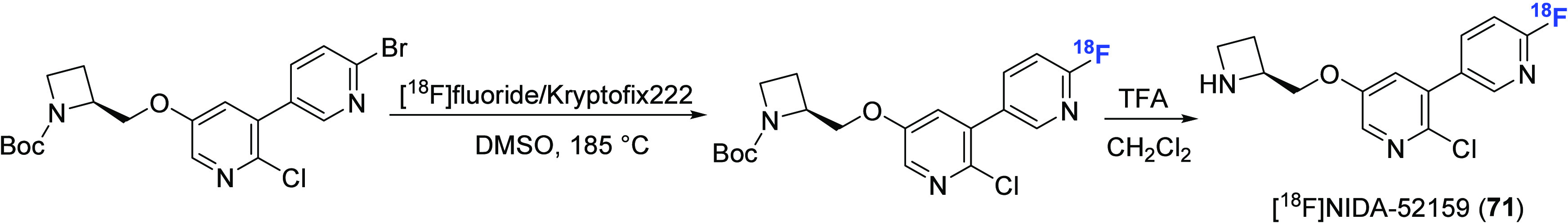
Representative radiofluorination method for the preparation of α4β2 nAChR-selective PET ligands.

The α4β2 subtype of nAChRs is a heteromeric receptor composed of α4 and β2 subunits, and it is the most abundant subtype of nAChRs in the brain.^167^ Due to their critical roles in a variety of neurological disorders such as nicotine addiction,^168,169^ AD,^170,171^ PD,^172,173^ and depression,^174,175^ α4β2 nAChRs have become one of the most extensively studied subtypes of nAChRs. PET imaging has emerged as a promising tool for measuring α4β2-nAChR levels in vivo, which could aid in disease diagnosis and the identification of patient subpopulations that would benefit from α4β2-nAChR-targeted therapy. Figures 16 and 17 provide an overview of representative α4β2 nAChR-selective PET ligands and selected radiosynthetic methods, respectively.

The first attempts to utilize PET studies for measuring α4β2-nAChRs involved the use of the radiolabeled nicotine, [^11^C]nicotine (**66**), on nonhuman primates, healthy human volunteers, and patients with AD.^176,177^ However, due to the nonspecific nature of [^11^C]nicotine (**66**) and its rapid metabolism, this approach was deemed unfavorable.^178,179^ To overcome these limitations, researchers developed two 3-pyridylether derivatives, namely, 2-[^18^F]F-A85380 (**67**, also known as 2-[^18^F]FA) and 6-[^18^F]F-A85380 (**68**) (Figure 16).^180^ While the derivatives 2-[^18^F]FA (**67**) and 6-[^18^F]F-A85380 (**68**) have shown unfavorable brain kinetics for quantitative kinetic modeling,^181,182^ the high affinity and specificity of 2-[^18^F]FA (**67**) (*K*_i_ = 0.05 nM) toward α4β2-nAChRs has allowed noninvasive visualization of these receptors in the human thalamus.^183^ In addition to the ^18^F-labeled nicotine derivatives, [^11^C]-methyl-3-(2-(*S*)-azetidinylmethoxy)pyridine ([^11^C]MA (**69**), *K*_i_ = 0.047 nM)^184^ has also been investigated as a tracer for α4β2- nAChR in PET imaging studies with rhesus monkeys.^185^

The majority of subsequent attempts to develop improved α4β2-nAChR PET ligands have focused on addressing the limitation of slow brain washout, as observed in the case of 2-[^18^F]FA (**67**), which precluded conventional kinetic tracer modeling and required up to 5-fold prolonged scanning times. Of note, Zhang et al. hypothesized that the slow brain kinetics of 2-[^18^F]FA (**67**) may be attributed to suboptimal lipophilicity and subsequently designed a library of derivatives with a broad range of lipophilicity. Two of these derivatives showed 2.5-fold improved binding potential compared to 2-[^18^F]FA (**67**) in rhesus monkeys, and suitable precursors were radiofluorinated to furnish radioligands [^18^F]NIDA-52158 (**70**) (*K*_i_ = 9 pM) and [^18^F]NIDA-52159 (**71**, Figure 17) (*K*_i_ = 4.9 pM), respectively.^186^ However, further studies are needed to evaluate the brain kinetics of these probes in order to understand their potential for use in PET imaging.

Additional studies led to the discovery of two epibatidine derivatives [^18^F]FPhEP (**72**) and [^18^F]F_2_PhEP (**73**) as potential tracers for α4β2-nAChRs (Figure 16). These compounds exhibited high to excellent in vitro affinity (*K*_i_ = 733 and 78 pM, respectively) toward the α4β2 subtype of nAChRs. However, although [^18^F]FPhEP (**72**) demonstrated superior brain kinetics compared to 2-[^18^F]FA (**67**), both ligands were not suitable for PET imaging applications, primarily due to limited in vivo specificity.^187,188^ Several other homoepibatidine derivatives have been developed as potential tracers for α4β2 in PET imaging. For example, the two enantiomers of [^18^F]flubatine ([^18^F]NCFHEB) (**74**) showed high binding affinity toward α4β2-nAChRs (*K*_i_ = 0.064–0.112 pM) but exhibited slow washout from the thalamus in monkey studies.^189,190^ The epibatidine derivatives, such as [^18^F]AZAN (**75**) and [^18^F]XTRA (**76**), have demonstrated slightly improved performance characteristics.^191^ In vivo baboon PET studies with [^18^F]AZAN (**75**) have shown high specific binding and a reduced study time to obtain quantitative information on α4β2-nAChR abundance.^192^ Similarly, [^18^F]XTRA (**76**) ([^18^F](−)-JHU86428) exhibited promising in vitro binding characteristics, including subnanomolar binding affinity and improved lipophilicity over that of 2-[^18^F]FA (**67**).^191^

Mukherjee and colleagues have made significant contributions to the field of α4β2-nAChRs PET imaging by developing several radioligands. Initially, they reported on the successful development of 5-(3-[^18^F]fluoropropyl)-3-(2-(*S*)-pyrrolidinylmethoxy)pyridine ([^18^F]nifrolidine (**77**)) and 2-[^18^F]fluoro-3-[2-((*S*)-3-pyrrolinyl)methoxy]pyridine ([^18^F]nifene (**78**)), which both possessed faster binding kinetics than previously reported agents and showed regional uptake patterns that corresponded with the known expression patterns of α4β2-nAChRs in the rat and monkey brains.^193,194^ Subsequently, 3-(2-(*S*)-azetidinylmethoxy)-5-(3′-[^18^F]fluoropropyl)pyridine ([^18^F]nifzetidine (**79**)) was designed to reduce off-target binding that was observed with [^18^F]nifrolidine (**77**) by substituting the pyrrolidine ring in the [^18^F]nifrolidine (**77**) structure with an azetidine ring. In vivo rhesus monkey PET imaging experiments using [^18^F]nifzetidine (**79**) revealed high levels of tracer uptake in the thalamus and extrathalamic regions, which are known to be α4β2-rich brain regions. Nonetheless, the slow kinetics of [^18^F]nifzetidine (**79**) necessitated a prolonged imaging time of greater than 3 h for quantitative PET studies.^195^ Further optimization led to the development of 3-(2-(*S*)-3,4-dehydropyrrolinylmethoxy)-5-(3′-[^18^F]fluoropropyl)pyridine ([^18^F]nifrolene (**80**)), which has a binding affinity for α4β2-nAChRs similar to that of 2-[^18^F]FA (**67**).^196^ In vivo rhesus monkey PET studies of [^18^F]nifrolene revealed the highest binding in the thalamus followed by regions of the lateral cingulated and temporal cortex and least binding in the cerebellum. Of note, the thalamus to cerebellum ratio in the monkey brain was >3 at 120 min, which was higher than that for [^18^F]nifrolidine and [^18^F]nifzetidine, suggesting promise of [^18^F]nifrolene as a new PET imaging agent for α4β2 nAChR.

A few promising α4β2-targeted PET ligands have advanced to clinical studies and have demonstrated encouraging results in their initial applications in humans. For instance, 2-[^18^F]FA (**67**) has been employed to detect the downregulation of α4β2-nAChRs in patients with AD and PD.^183,197−199^ Furthermore, it has been utilized to evaluate the effects of nicotine-induced upregulation of nAChRs in active smokers and during smoking cessation.^200^ These findings have led to further clinical investigations using 2-[^18^F]FA (**67**) to assess changes in cerebral nAChRs in healthy nonsmokers, ex smokers, and heavy and light situational smokers (NCT01038245 and NCT01046513). Similar to 2-[^18^F]FA (**67**), first in human studies have indicated that [^18^F]nifene (**78**) was a reliable and reproducible radiotracer to visualize α4β2-nAChRs in the human brain. Its favorable brain kinetics enabled kinetic tracer modeling with substantially reduced scan times, which is particularly suitable for use in vulnerable populations.^201^ Furthermore, the enantiomerically pure α4β2-nAChR PET radioligand (−)-[^18^F]flubatine (**81**) exhibited high in vivo stability, fast brain kinetics, and rapid uptake and equilibration between free and receptor-bound tracer in the human brain, making it a valuable tool for imaging α4β2-nAChRs in neuropsychiatric disorders.^202^ Another probe [^18^F]AZAN (**75**) was also evaluated in humans, and a 90 min PET scan with [^18^F]AZAN (**75**) was sufficient for performing quantitative analysis of α4β2-nAChR in the living human brain.^203^ [^18^F]AZAN (**75**) has been used to study the relationship between the extent of α4β2-nAChRs occupancy induced by varenicline and the magnitude of dopamine release following nicotine use.^204^ Lastly, [^18^F]XTRA (**76**), a radioligand that exhibits relatively high binding potentials in extrathalamic regions of the baboon brain, has been successfully utilized for quantitative human neuroimaging of the extrathalamic α4β2-nAChR. This finding provides further support for the concept that α4β2-nAChR may play an important role in age-dependent remodeling of the human brain.^205,206^

Overall, a large number of nAChR PET radioligands have been developed, but many of them are still in preclinical investigation or in clinical evaluation for visualizing cerebral nAChRs in neurodegenerative diseases. Recent interest has grown in the potential of nAChRs for diagnosing and treating other neurological diseases beyond neurodegenerative diseases.^207^ This renewed interest may stimulate further development and application of nAChR PET radioligands for these purposes.

The properties and molecular imaging results of α4β2 nAChR-selective PET ligands discussed in this section are presented in Table 6.

**Table 6:**
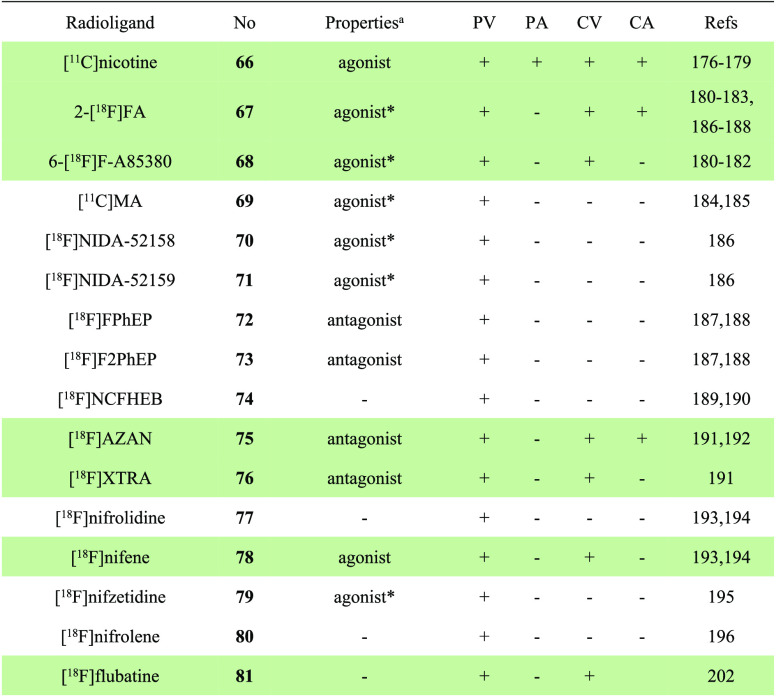
Properties and Molecular Imaging Results of α4β2 nAChR-Selective PET Ligands^176−196,202^

a The definition of antagonist or agonist is based on literature reports. PV, preclinical validation; PA, preclinical application; CV, clinical validation; CA, clinical application. The light green shading indicates the investigation stage of translation into human use.

* Predicted from A85380.

Apart from vast application in identifying the regional nAChR and mAChR availability under normal conditions and their alteration under disease conditions and/or following drug exposure, PET imaging can also assess endogenous ACh concentrations, which are quantified by regional differences in receptor occupancy. For instance, α4β2-nAChR PET radioligand (−)-[^18^F]flubatine (**81**) has demonstrated sensitivity to changes in ACh levels caused by the acetylcholinesterase inhibitor physostigmine.^208^ Subsequently, Hillmer and co-workers used (−)-[^18^F]flubatine (**81**) and M1-preferring radioligand [^11^C]LSN3172176 (**24**) to measure baseline variation in ACh concentration across regions in the living human brain, offering a novel approach to estimate potential ACh imbalances in clinical use.^209^

## 4 Perspectives and Conclusion

The development of PET tracers that selectively target cholinergic receptors has sparked a vibrant and rapidly advancing research field, holding immense promise for deepening our understanding of these receptors and their associated disorders. Despite a number of cholinergic receptor-targeted PET tracers having been disclosed, many of them failed in preclinical investigation. The most common challenge resides in flow-dependent tracer accumulation, which confounds accurate and reliable quantification of cholinergic receptors in tissues of interest. Other challenges include metabolic instability, limited binding specificity, and inability to cross the BBB. Therefore, it is not surprising that future cholinergic receptor-targeted PET tracer development should combine conventional medicinal chemistry with rational pharmacological design in the mechanism of action including orthosteric, allosteric, and bitopic binding to optimize ligand characteristics. Further, although several cholinergic receptor-targeted PET tracers have been advanced into clinical evaluation, the vast majority of them focus on imaging of cerebral cholinergic receptors. Given the advent of whole-body PET, a deeper understanding of cholinergic receptors in the periphery might be of interest as a complementary finding to CNS studies. Ultimately, the development and application of cholinergic receptor-targeted PET tracers will have a significant impact on the diagnosis, treatment, and management of cholinergic receptor-related disorders.

## Abbreviations Used

Aβ: amyloid beta plaque
ACh: acetylcholine
AChR: acetylcholine receptor
AD: Alzheimer’s disease
ARG: autoradiography
BBB: blood–brain barrier
BP_ND_: non-displaceable binding potential
BP: binding potential
cAMP: cyclic adenosine monophosphate
ChAT: choline acetyltransferase
CNS: central nervous system
CoA: coenzyme A
DEX: dexetimide
G-protein: GTP binding regulatory protein
GPCR: G-protein-coupled receptor
GTP: guanosine triphosphate
HD: Huntington’s disease
%ID/g: percentage injected dose per gram of tissue
mAChR: muscarinic acetylcholine receptor
nAChR: nicotinic acetylcholine receptor
NHP: nonhuman primate
NMPB: *N*-methyl-4-piperidylbenzilate
PD: Parkinson’s disease
ALS: amyotrophic lateral sclerosis
MS: multiple sclerosis
PET: positron emission tomography
Pgp: P-glycoprotein
PNS: peripheral nervous system
QNB: quinuclidinyl benzilate
RCY: radiochemical yield
SPECT: single-photon emission computed tomography
SUV: standard uptake value
SU*V*_max_: maximum standardized uptake value
TRB: (+)-2α-tropanyl benzilate
